# Synthesis and Luminescence of Optical Memory Active Tetramethylammonium Cyanocuprate(I) 3D Networks

**DOI:** 10.3390/ma12081211

**Published:** 2019-04-12

**Authors:** Aaron D. Nicholas, Rebeka M. Bullard, Amelia M. Wheaton, Michaela Streep, Victoria A. Nicholas, Robert D. Pike, Howard H. Patterson

**Affiliations:** 1 Department of Chemistry, University of Maine, Orono, ME 04469, USA; rebeka.bullard@maine.edu (R.M.B.); victoria.nicholas@maine.edu (V.A.N.); howardp@maine.edu (H.H.P.); 2 Department of Chemistry, College of William and Mary, Williamsburg, VA 23187-8795, USA; amwheaton@wisc.edu (A.M.W.); mestreep@email.wm.edu (M.S.); rdpike@wm.edu (R.D.P.)

**Keywords:** luminescence, crystallography, optical memory, charge transfer, copper cyanide

## Abstract

The structures of three tetramethylammonium cyanocuprate(I) 3D networks [NMe_4_]_2_[Cu(CN)_2_]_2_•0.25H_2_O (**1**), [NMe_4_][Cu_3_(CN)_4_] (**2**), and [NMe_4_][Cu_2_(CN)_3_] (**3**), (Me_4_N = tetramethylammonium), and the photophysics of **1** and **2** are reported. These complexes are prepared by combining aqueous solutions of the simple salts tetramethylammonium chloride and potassium dicyanocuprate. Single-crystal X-ray diffraction analysis of complex **1** reveals {Cu_2_(CN)_2_(*μ*_2_-CN)_4_} rhomboids crosslinked by cyano ligands and D_3h_ {Cu(CN)_3_} metal clusters into a 3D coordination polymer, while **2** features independent 2D layers of fused hexagonal {Cu_8_(CN)_8_} rings where two Cu(I) centers reside in a linear C_∞v_ coordination sphere. Metallophilic interactions are observed in **1** as close Cu⋯Cu distances, but are noticeably absent in **2**. Complex **3** is a simple honeycomb sheet composed of trigonal planar Cu(I) centers with no Cu^…^Cu interactions. Temperature and time-dependent luminescence of **1** and **2** have been performed between 298 K and 78 K and demonstrate that **1** is a dual singlet/triplet emitter at low temperatures while **2** is a triplet-only emitter. DFT and TD-DFT calculations were used to help interpret the experimental findings. Optical memory experiments show that **1** and **2** are both optical memory active. These complexes undergo a reduction of emission intensity upon laser irradiation at 255 nm although this loss is much faster in **2**. The loss of emission intensity is reversible in both cases by applying heat to the sample. We propose a light-induced electron transfer mechanism for the optical memory behavior observed.

## 1. Introduction

Researchers continue to be attracted to copper(I) based coordination polymers which display a number of desirable photophysical behaviors including high emission quantum yields [1], emission energy tunability [2,3,4,5], thermally activated delayed phosphorescence [6,7,8], photochromism [9], thermochromism [10,11,12], vapochromism [13,14,15], and optical memory [16,17]. Most recently we have been interested in the optical memory behavior of these materials [16,18]. Materials that display optical memory are able to undergo a reversible change in emission upon laser irradiation such as reduction in emission intensity. Since the loss of intensity is localized, it is possible to “write” onto these materials by turning “off” emission in select areas via laser irradiation. This reduction of emission intensity is maintained at low temperatures. Simply heating the area results in recovery of the emission back to the original intensity.

The mechanism by which optical memory occurs is difficult to predict and is dependent on a number of variables including chemical composition and structure [16,17,19,20,21,22,23]. For example, CuSCN(3-BrPy)_2_ (3-BrPy = 3-bromopyridine) is composed of 1D metal/thiocyanate chains decorated with halopyridine ligands [4,16]. This complex exhibits luminescence behavior through a metal thiocyanate-to-pyridine charge transfer whereby yellow emission is observed at 538 nm. Upon laser irradiation at 266 nm the halopyridine undergoes a C-Br bond length increase during which the halide atom is captured by a neighboring thiocyanate, forming a halide bridge. This halogen-bridged structure resides in a metastable energy well which prevents relaxation of the complex back to the ground state, effectively quenching the emission in the “off” state. Heating the sample to 298 K provides ample energy to the system permitting breakage of the halogen-bridge, returning the system back to the ground state. Metal-metal redox, isomerization, and triplet annihilation mechanisms, with special attention by our group on d^10^ cyanometallates, have also been reported [17,19,24,25,26,27,28,29,30].

In an effort to expand our fundamental understanding of these materials we have been focusing on optical memory behavior in systems containing the ion pairs CuCN_2_^−^ and tetramethylammonium (Me_4_N^+^). The quaternary ammonium ion Me_4_N^+^ is not redox active and, therefore, is unable to participate in interionic charge transfer behavior, leaving only the CuCN_2_^−^ subunits as the emissive species. We have observed interesting emissive behavior in analogous AuCN_2_^−^/AgCN_2_^−^ materials but have not as yet investigated their optical memory potential [31]. Herein, we report on the structure and optical memory of two new 3D networks [NMe_4_]_2_[Cu(CN)_2_]_2_•0.25H_2_O (**1**) and [NMe_4_][Cu_3_(CN)_4_] (**2**). Despite similar preparative conditions, these complexes contain distinctly different structural topologies and luminescence behavior. Complex **1**, which emits bright green under UV light irradiation at room temperature, displays thermochromic behavior, emitting blue upon cooling. Conversely, **2** emits bright blue under black light irradiation at all temperatures and displays no thermochromic behavior. While both **1** and **2** exhibit optical memory behavior upon laser irradiation at 255 nm, their rates of emission intensity reduction are dramatically different. Our findings demonstrate that in the case of **1** the presence of singlet emission lessens the rate of intensity reduction upon laser irradiation. We believe our findings are beneficial in the rational design of optical memory active complexes given the difficulty in predicting their emission reduction/recovery mechanism.

## 2. Materials and Methods

### 2.1. General

All reagents were purchased from Aldrich (St. Louis, MO, USA), Acros (Geel, Antwerp, Belgium), or American Element (Los Angeles, CA, USA) and were used as received. Infrared spectra were collected on solid samples at 298 K between 450 cm^−1^ and 4000 cm^−1^ using a Perkin Elmer FT-IR Spectrum Two equipped with a universal attenuated total reflectance (UATR) accessory and LiTaO_3_ MIR detector. Diffuse reflectance spectra were collected on solid samples at 298 K. The light source was a Mikropack DH-2000 deuterium and halogen light source coupled with an Ocean Optics USB4000 detector. Scattered light was collected with a fiber optic cable. Spectra were referenced with MgSO_4_. Data was processed using SpectraSuite 1.4.2_09. Compounds were analyzed for their Cu content via flame atomic absorption spectroscopy (AAS) using a Perkin Elmer AAnalyst 700 instrument. Samples were dissolved in 7% nitric acid (by weight) and then diluted in water to a concentration of 0.100 g/L. All samples were measured three times with a reported average value. CHN analysis services were performed by Atlantic Microlab in Atlanta, Georgia.

### 2.2. Synthesis

*[Me_4_N]_2_[Cu(CN)_2_]_2_•0.25H_2_O*, **1**. Me_4_NCl (245 mg, 2.24 mmol), KCN (96 mg, 1.48 mmol), and CuCN (133 mg, 1.48 mmol) were stirred in 10 mL of DI water and heated to 100 °C for 3 hrs. The blue emissive suspension was allowed to cool to room temperature overnight and then cooled to 5 °C for seven days. The off-white suspension (120 mg, 0.315 mmol) was filtered and washed with DI water, 95% ethanol, and diethyl ether. Measured AAS Cu content = 43.8% (theoretical = 45.2%). CHN Analysis for C_14_H_24.50_Cu_4_N_8_O_0.25_: Theoretical (%): C 29.86, H 4.38, N 19.90; experimental (%): C 29.92, H 4.24, N 20.02.

*[Me_4_N][Cu_3_(CN)_4_]*, **2**. Me_4_NCl (45 mg, 0.41 mmol), KCN (80 mg, 1.23 mmol), and CuCN (110 mg, 1.23 mmol) were stirred in 10 mL of DI water and heated to 100 °C overnight. The off-white, blue-emissive suspension (54 mg, 0.15 mmol) was filtered hot and washed with DI water, 95% ethanol, and diethyl ether. Measured AAS Cu content = 51.2% (theoretical = 51.7%). CHN Analysis for C_16_H_24_Cu_6_N_10_: Theoretical (%): C 26.05, H 3.28, N 18.99; experimental (%): C 26.20, H 3.14, N 19.22.

*[Me_4_N][Cu_2_(CN)_3_]*, **3**. Hexagonal plate crystals of **3** were occasionally found in crystallized batches of **2**. No conditions were found under which this complex could be reliably formed. 

### 2.3. X-Ray Analysis

All measurements were made using microfocus Cu K*α* (**1** and **2**) or sealed tube Mo K*α* (**3**) radiation on a Bruker-AXS three-circle Apex DUO diffractometer, equipped with a SMART Apex II CCD detector. Initial space group determination was based on a matrix consisting of 90 or 36 frames. The data were reduced using SAINT+ [32,33], and empirical absorption correction applied using SADABS [34]. A crystal was mounted on a glass fiber, and a full dataset was collected at 100 K or 296 K. The structures were solved using intrinsic phasing. Least-squares refinement for all structures was carried out on *F*^2^. The non-hydrogen atoms were refined anisotropically. Hydrogen atoms were located in the Fourier difference map and then allowed to refine isotropically. The hydrogen atoms on the 0.25 occupancy water molecule of **1** were not located and were not placed in the final structure. Structure solutions were carried out using SHELX [35] and refinements were performed using the ShelXle program [36]. Crystal data and structural refinement parameters can be found in Table 1.

### 2.4. Photophysical Studies

Steady-state luminescence scans were collected between 298 K and 78 K. Spectra were recorded using a Model Quantamaster-1046 photoluminescence spectrophotometer from Photon Technology International using a 75 W xenon arc lamp combined with two excitation monochromators and one emission monochromator. A photomultiplier tube at 800 V was used as the emission detector. The solid samples were mounted on a copper plate using non-emitting copper-dust high vacuum grease. All scans were collected under vacuum with a Janis ST-100 optical cryostat. Low temperature scans used liquid nitrogen for scans down to 78 K. Laser irradiation experiments were performed with a Opolette Model 355II and UV tunable pulse laser using a Nd:Yag laser pump. Focusing lenses were initially adjusted prior to irradiation to maximize emission intensity for each compound. Samples were irradiated at 78 K at five-minute intervals. Recovery measurements were accomplished by allowing samples to reach thermal equilibrium at 298 K before being cooled back to low temperatures.

### 2.5. Molecular Modelling

To investigate the luminescence properties of **1** and **2** we have performed density functional theory (DFT) and time-dependent density functional theory (TD-DFT) calculations using Gaussian 16 software (Gaussian Inc.) with the University of Maine Advanced Computing Group [37]. Initially, ground state calculations were performed on models using both the B3LYP [38,39] level of theory with the modified scalar-relativistic effective core potential (ECP) basis set SDD [40,41,42,43,44] as implemented in the software and the M06 meta-hybrid functional [45,46] level of theory with the CEP-31G(d) basis set [47,48]. We have developed models for **1** and **2** using the X-ray data as the initial input. Calculations were performed on an anionic subunit of **1** with formula [Me_4_N]_4_[Cu_14_(CN)_27_] and of **2** with formula [Me_4_N]_2_[Cu_8_(CN)_14_]. The models of **1** and **2** maintain the local geometry of the Cu(I) coordination spheres while, in the case of **1**, account for the close measured Cu⋯Cu distances. Ground state parameters (Supplementary Materials) show better agreement to experimental values using the M06/CEP-31G(d) method, especially in the case of close Cu⋯Cu distances, and so this method/basis set was selected for TD-DFT calculations. Deviations in bond lengths/angles are considered minor and are attributed to lack of crystal packing. Because of the agreement between experimental observations and theoretical values we believe our models accurately describe the photophysical behavior of **1** and **2**. Isodensity representations of molecular orbitals were visualized using the Avogadro 1.2.0 software program [49].

## 3. Results

### 3.1. Structural Studies

**Figure 1:**
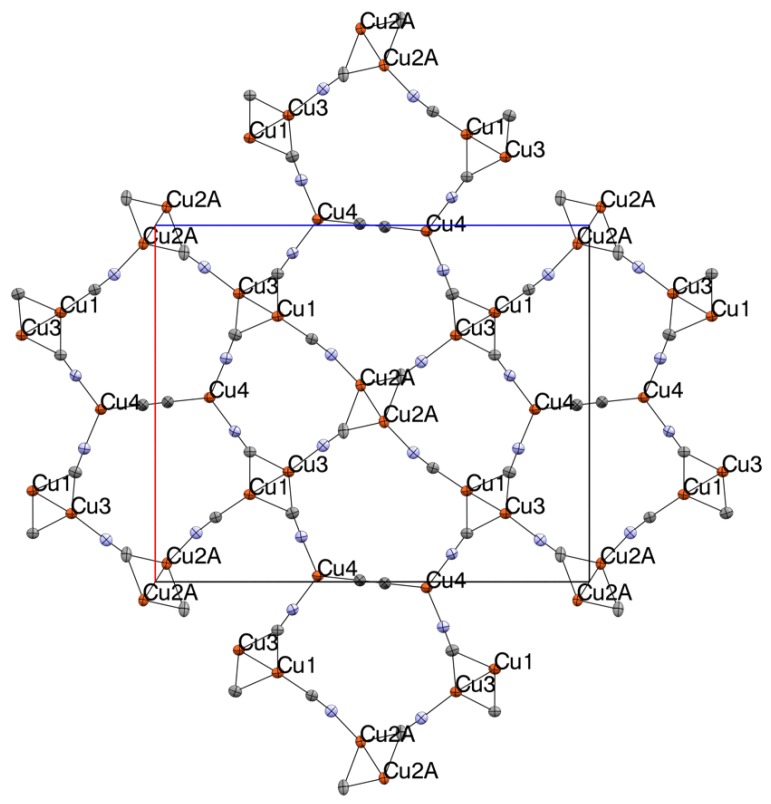
Thermal ellipsoid (50% probability) packing diagram of **1**, projected along the *b*-axis, showing network formation. Me_4_N^+^ cations omitted for clarity. Cu orange, N blue, C grey. Selected bond lengths and angles (X = disordered C/N): Cu1-X1 1.960(4), Cu1-X3 2.236(4), Cu1-X4 2.089(4), Cu1-X8 1.963(4), Cu1-Cu3 2.5146(8), Cu2A-X1 2.005(6), Cu2A-C2 2.037(6), Cu2A-C2 2.298(9), Cu2A-X6 1.981(6), Cu2A-Cu2A 2.435(12), Cu3-N2 2.023(4), Cu3-C3 2.007(4), Cu3-C4 2.077(4), Cu4-X5 1.904(5), Cu3-X7 1.988(4), Cu4-N3 1.923(4), Cu4-N4 1.913(4), X1-X1 1.161(6)N2-C2 1.145(6), N3-C3 1.151(6), N4-C4 1.152(5), X5-X5 1.138(10), X6-X6 1.180(8), X7-X7 1.167(8), X8-X8 1.151(8), X1-Cu1-N8 114.60(15), X1-Cu1-X8 114.60(15), X1-Cu1-C4 111.26(15), X8-Cu1-C4 113.10(15), X1-Cu1-C3 111.54(15), X8-Cu1-C3 103.14(15), C4-Cu1-C3 102.20(16), X6-Cu2A-X1 111.0(3), X6-Cu2A-C2 110.1(3), X1-Cu2A-C2 110.4(3), X6-Cu2A-C2 104.6(3), X1-Cu2A-C2 108.7(3), C2-Cu2A-C2 112.0(3), X7-Cu3-C3 110.74(16), X7-Cu3-N2 105.74(14), C3-Cu3-N2 113.45(16), X7-Cu3-C4 106.79(15), C3-Cu3-C4 111.08(17), N2-Cu3-C4 108.69(15), X7-Cu3-Cu1 123.84(11), X5-Cu4-N4 120.58(18), X5-Cu4-N3 119.12(18), and N4-Cu4-N3 120.28(15).

The tetramethylammonium cyanocuprate(I) compounds were readily crystallized from aqueous solutions. Details of the X-ray experiments and crystal data are summarized in Table 1. Selected bond lengths and bond angles are given in Figures 1, 3 and Figure 4, and in the Supplementary Materials. While the Me_4_N^+^ cyanocuprate(I) complexes herein are the first reported salts of this cation, cyanocuprates are known for other cations including alkylammonium [50,51,52,53,54,55,56], phosphonium ions [57], alkali metal ions [58], and copper(II) ions [54,55,59,60,61,62,63,64,65], and protonated water or methanol [60,66]. In all cases, 2D or 3D anionic networks result from bridging of the cyano ligands. In most cases, copper atoms are 3-coordinate, but 2- and/or 4-coordination are also observed on occasion.

The structure **1** solved in the orthorhombic space group *Pnma*. The crystallographically independent unit consists of four Cu atoms, four full and four half cyano groups, and four half Me_4_N^+^ ions, summing to the formula: (Me_4_N)_2_[Cu_4_(CN)_6_]. As shown in Figure 1, complex **1** is composed of a series of {Cu_2_(CN)_4_(*μ*_2_-CN)_2_} rhomboids crosslinked by cyano ligands and D_3h_ {Cu(CN)_3_} metal clusters to form 2D planar sheets. The {Cu_2_(CN)_4_(*μ*_2_-CN)_2_} rhomboids, which feature 4-coordinate Cu centers with close Cu^…^Cu interactions, have been seen in several related cyanocuprate structures [53,55,61,62,63,65]. The 2D sheets are further crosslinked via perpendicular cyano ligands emanating from the rhomboids to form a 3D coordination network composed of large five-sided channels bounded by three rhomboids and two D_3h_ units. All Me_4_N^+^ ions lie within these channels. 

Two inversion centers are present in **1**, being located between the Cu2A atoms and the trigonal planar Cu4 atoms. These result in a mirror plane through the unit cell. Overall, there is much disorder throughout the structure. First, (half-independent) cyanide ligands C5/N5, C6/N6, C7/N7, and C8/N8 are each disordered across special positions (symmetrically disordered), requiring 50:50 occupancy of C and N in each case. Fully independent cyano C1/N1 also shows C/N disorder, but without a special position (non-symmetrically disordered); nevertheless, refinement of the carbon and nitrogen occupancies at both sites resulted in nearly 50:50. Cu2 is found in two closely adjacent sites, having about 70:30 occupancy. A pair of Cu2 atoms forms a rhomboid dimer around a special position. Finally, there is disorder in one of the three half Me_4_N^+^ ions (C16, C17, C18) which, itself, shows interaction with the partially occupied (0.25) water molecule. Significant metallophilic interactions are observed across each of the rhombic centers via short Cu⋯Cu distances: 2.436(12) Å (Cu2^…^Cu2) and 2.5146(8) Å (Cu1^…^Cu3). The site disorder in Cu2 noted above makes the Cu^…^Cu distance less certain in this case. The van der Waals (VDW) sum for Cu⋯Cu is typically considered 2.8 Å [67]. Contraction by the Cu⋯Cu interaction causes significant broadening of the rhombic C-Cu-C angles, falling between 102.19° and 111.06°. The Cu-C-Cu angles of the *μ*_2_-CN are between 72.47° and 74.26°. The D_3h_ metal cluster geometry is undistorted. Measured Cu-C and Cu-N bond lengths are unremarkable throughout, with generally longer bond lengths for *μ*_2_-CN ligands. 

**Figure 2:**
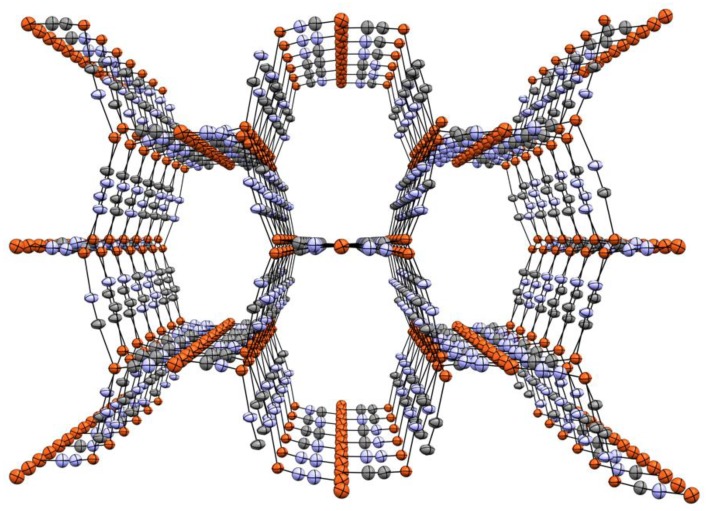
Thermal ellipsoid (40% probability) packing diagram of **2** at 295 K along the *c*-axis, showing stacked elongated {CuCN}_8_ hexagons. Me_4_N^+^ cations omitted for clarity. Cu orange, N blue, C grey.

At 295 K compound **2** crystallizes in the monoclinic *C2/c* space group, Figure 2. The unit cell holds only one and a half independent Cu atoms and two CNs, both of which are non-symmetrically disordered (63:37 and 56:44, respectively). The half-independent Cu(I) center is 2-coordinate and lies on a linear C_∞v_ coordination site, while the other is 3-coordinate and roughly trigonal planar (D_3h_). All Cu atoms are connected by *μ*_2_-bridging cyano ligands, forming simple 2D sheets composed of elongated fused hexagonal {Cu_8_(CN)_8_} rings. This and closely related motifs have been observed previously [51,55,58,59]. The rings are stacked such that trigonal Cu2 lies almost directly above linear Cu1, the shortest interplanar Cu⋯Cu distance being 3.076 Å and, thus, without metallophilic interactions. Again, two crystallographic inversion centers are observed, one at the macrocyclic ring center and the other at Cu1, shared between adjacent rings. Only one independent, two-site disordered Me_4_N^+^ ion is present, located between the sheets centered on openings in the macrocycles. 

**Figure 3:**
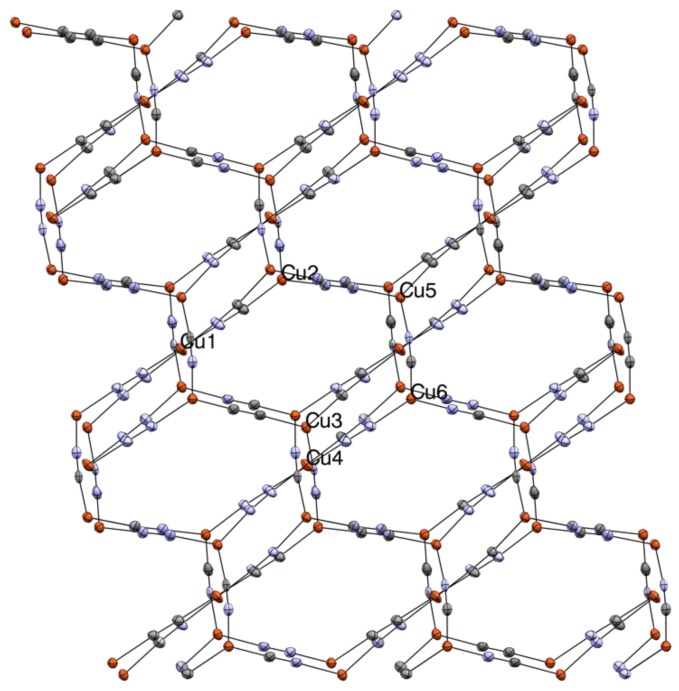
Thermal ellipsoid (50% probability) packing diagram of **2** at 100 K along the *c*-axis, showing staggered {CuCN}_8_ hexagons. Me_4_N^+^ cations and minor occupancy Cu atoms omitted for clarity. Cu orange, N blue, C grey. Selected bond lengths and angles (X = disordered C/N): Cu1-X1 1.857(4), Cu1-X2 1.847(4), Cu1-Cu5 2.9370(9), Cu2-X2 1.931(4), Cu2-X3 1.924(4), Cu2-X4 1.946(4), Cu2-CuA 3.0512(9), Cu3-X4 1.912(4), Cu3-X5 1.931(4), Cu3-X8 1.944(4), Cu4-X5 1.850(4), Cu4-X6 1.844(4), Cu4-Cu6 2.9971(9), X1-X1 1.154(6), Cu5-X3 1.947(4), Cu5-X6 1.927(4), Cu5-X7 1.915(4), Cu6-X1 1.944(4), Cu6-X7 1.933(4), Cu6-X8 1.922(4), X1-X1 1.157(5), X2-X2 1.155(6), X3-X3 1.158(6), X4-X4 1.166(6), X5-X5 1.160(6), X6-X6 1.167(6), X7-X7 1.175(6), X8-X8 1.156(6), X1-Cu1-X2 177.68(18), X1-Cu6-X8 123.78(17), X2-Cu2-X3 125.06(17), X2-Cu2-X4 118.29(17), X3-Cu2-X4 116.27(17), X3-Cu5-X6 116.85(16), X3-Cu5-X7 116.54(16), X4-Cu3-X5 122.42(17), X4-Cu3-X8 119.31(16), X5-Cu3-X8 117.93(16), X5-Cu4-X6 178.72(18), X6-Cu5-X7 126.24(16), X7-Cu6-X1 116.31(16), and X7-Cu6-X8 119.55(16).

Interestingly, upon cooling **2** undergoes a phase change, albeit without significant structural alterations. The new phase, Figure 3, is in the triclinic space group *P-1*. As is the case with the ambient temperature phase, this low temperature phase has two distinct metal coordination geometries, 2-coordinate C_∞v_ and 3-coordinate D_3h_. However, the unit cell contains six crystallographically independent Cu atoms, two of which are two-coordinate and four which are 3-coordinate. There are eight independent CN groups, all of which are non-symmetrically C/N disordered. Occupancies vary from about 60:40 to 72:38. Two independent Me_4_N^+^ ions are also observed, one of which is disordered. As was the case at ambient temperature, 2D sheets of elongated fused hexagonal {Cu_8_(CN)_8_} rings are present. Also unchanged from room temperature is the staggering of the ring placements between layers with 2-coordinate and 3-coordinate copper centers lying roughly atop one another with the Me_4_N^+^ ions again located between the sheets centered on openings in the macrocycles. As was the case at 295 K, no metallophilic interactions are observed, with the inter-sheet Cu⋯Cu distances being close to 3 Å. 

**Figure 4:**
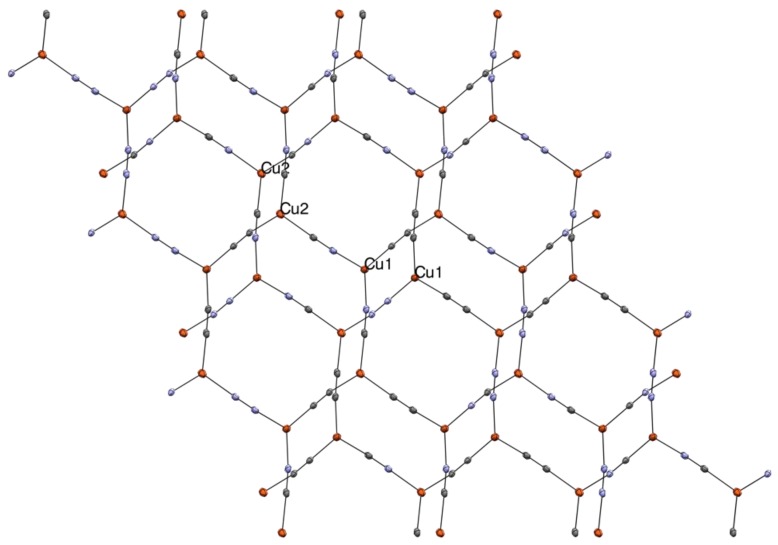
Thermal ellipsoid (50% probability) packing diagram of **3**, projected along the *c*-axis, showing network formation. Me_4_N^+^ cations omitted for clarity. Cu orange, N blue, C grey. Selected bond lengths and angles (X = disordered C/N): Cu1-X1 1.9311(17), Cu1-X2 1.9485(16), Cu1-X3 1.9421(16), Cu2-X1 1.9398(17), Cu2-X2 1.9656(16), Cu2-X3 1.9645(16), X1-X1 1.170(2), X2-X2 1.169(2), X3-X3 1.168(2), X1-Cu1-X2 120.90(6), X1-Cu1-X3 121.44(6), X2-Cu1-X3 117.17(6), X1-Cu2-X2 119.63(6), X1-Cu2-X3 120.28(6), and X2-Cu2-X3 114.38(6).

Compound **3** crystallized as colorless, non-emissive hexagonal plates in some of the aqueous crystallizations of compound **2**. The structure **2** solved in the triclinic space group *P–1.* The crystallographically independent unit consists of two Cu atoms and three non-symmetrically disordered cyano groups with nearly 50:50 C and N occupancies at each position, and a single ordered Me_4_N^+^ ion, summing to the formula: (Me_4_N)[Cu_2_(CN)_3_]. As shown in Figure 4, complex **3** forms simple hexagonal 2D sheets with no cyano bridging or cuprophilic interactions. This is a very common anionic network arrangement in cyanocuprates [52,53,54,55,57,58,60,63,65,66]. The sum of the angles around Cu1 and Cu2 are 359.51° and 354.59°, respectively. The slightly smaller angle sum for Cu2, is the result of a very weak long-range interaction (2.709 Å) with C1/N1 in an adjacent sheet. The Me_4_N^+^ ions lie centered over the hexagonal Cu_6_(CN)_6_ openings in each sheet with one Me group pointing into this opening.

### 3.2. Infrared Characterization

The dominant vibrational features of **1** and **2** are the cyanide and tetramethylammonium stretches. The IR vibrational spectra are shown in Figure S5 (Supplementary Materials). The vibrational modes of the Me_4_N^+^ cation are clearly observed for the δ_as_(C-H) at 1478 cm^−1^ and the ⱱ_as_(C-N) at 950 cm^−1^ [68]. The ⱱ_s_(C≡N) of cyanometallates are generally observed between 2040 cm^−1^ and 2150 cm^−1^. In the case of complex **1** this C≡N stretch is split with a peak at 2114 cm^−1^, 2070 cm^−1^, and a shoulder at 2094 cm^−1^, indicating inequivalent cyanide ligands within the crystal network. This splitting is in agreement with the structural data of **1** which show two types of cyanides: those that bridge individual {Cu_2_(CN)_4_]^2−^ rhomboids and those that bridge a {Cu_2_(CN)_4_]^2−^ rhomboid with a trigonal planar Cu(I) center. We have established in other systems that the bidentate nature of the *μ*^2^-CN decreases the cyanide bond order, lowering the vibrational frequency in comparison to the bridging cyanide [31]. Thus, for **1**, we assign the ⱱ_s_(C≡N) at 2114 cm^−1^ and 2070 cm^−1^ to the bridging *μ*^1−^ and *μ*^2−^CN group, respectively. For **2,** the cyanide stretches are shifted to higher energies with peaks at 2116 cm^−1^ and 2138 cm^−1^. In the case of **2**, X-ray studies show that the Cu(CN)_2_^−^ subunits exist in both linear and bent conformations. The linear Cu(CN)_2_^−^ coordination sphere consists of a Cu^+^ center coordinated to *two* CN^−^ ligands while in the bent coordination the Cu^+^ center is coordinated to *three* CN^−^ ligands. We argue that the increased electron density surrounding the three coordinate Cu^+^ center drives the stretching frequency of the CN band to lower energies in comparison to those of the two coordinate Cu^+^ center.

### 3.3. Photophysical Studies

**Figure 5:**
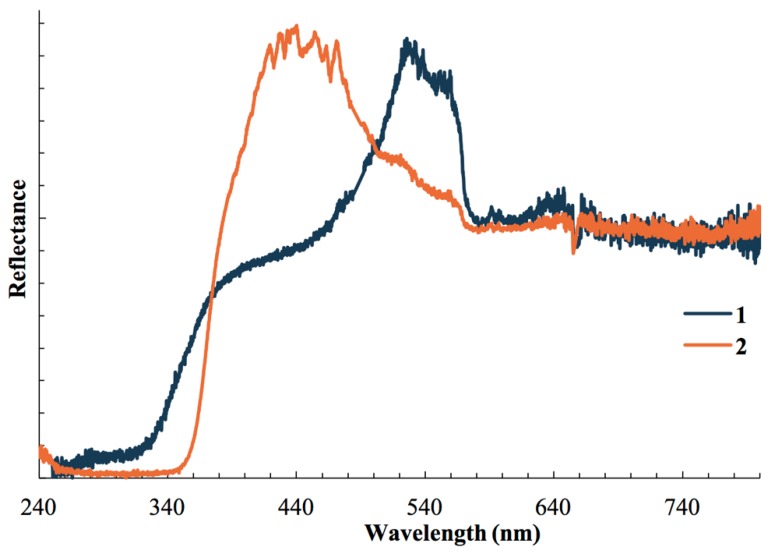
Diffuse reflectance spectra of solid samples of **1** and **2** at 298 K.

Solid samples of **1** and **2** both present themselves as off-white under room light. We have performed diffuse reflectance measurements of these complexes at 298 K, shown in Figure 5. All compounds absorb strongly in the UV region, with absorbance for **1** extending into the visible due to the presence of two absorption bands, one at 290 nm and 450 nm. The absorption edge of **1** (2.64 eV) is lower in energy compared to that of **2** (3.25 eV). Emission bands are observed for both complexes in the DRS spectra. We tentatively assign the absorption bands to a variation of metal-to-ligand charge transfer (MLCT) which we have observed in other cyanocuprate(I) complexes that lack electron-accepting cations [17,30,69,70,71]. In the case of **1** we ascribe the lower energy absorption edge to lower ground and excited state energies due to metallophilic interactions observed in the crystal structure.

**Figure 6:**
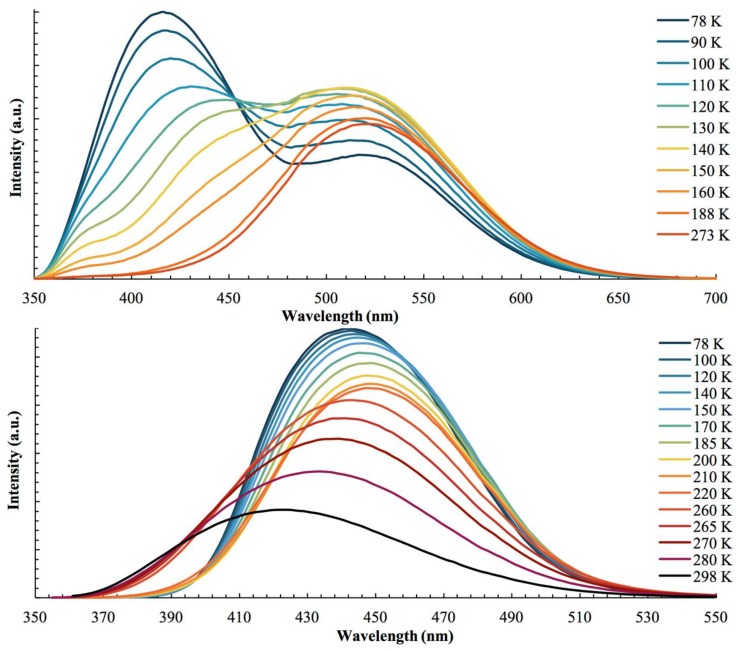
Luminescence spectra of **1** (top) and **2** (bottom) between 78 K and 298 K. The emission was measured at *λ*_ex_= 315 nm and *λ*_ex_ = 330 nm for **1** and **2**, respectively.

Despite sharing similar cation and anion subunits, complexes **1** and **2** display notably different photophysical behaviors. Variable temperature luminescence spectra between 298 K and 78 K for these complexes are summarized in Table 2 and shown in Figure 6. At room temperature complex **1** intensely emits green, with a maximum peak at 524 nm. Cooling the sample to 78 K results in the formation of an intense emission band at 416 nm, while the emission band at 524 nm is decreased in intensity. At 298 K the 524 nm emission is associated with a triplet excited state with a lifetime of 63 *μ*s. At 78 K the 524 nm emission decay rate is decreased to 0.31 *μ*s, while the new 416 nm band has a decay rate of 78 ns. These lifetime measurements indicate that **1** is a dual singlet/triplet emitter for which phosphorescence is dominant at high temperatures while fluorescence becomes dominant at low temperatures. This singlet/triplet behavior has been reported in other CuCN-based complexes [72,73]. At 298 K compound **2** displays a broad emission band centered at 423 nm. Cooling the sample red shifts this band to 450 nm until 130 K, from which point further cooling causes a blue shift back to higher energies, reaching 440 nm at 78 K. Lifetime measurement of **2** shows triplet emission at all temperatures (*τ*_298 K_ = 6.3 *μ*s, *τ*_78 K_ = 33 *μ*s).

**Figure 7:**
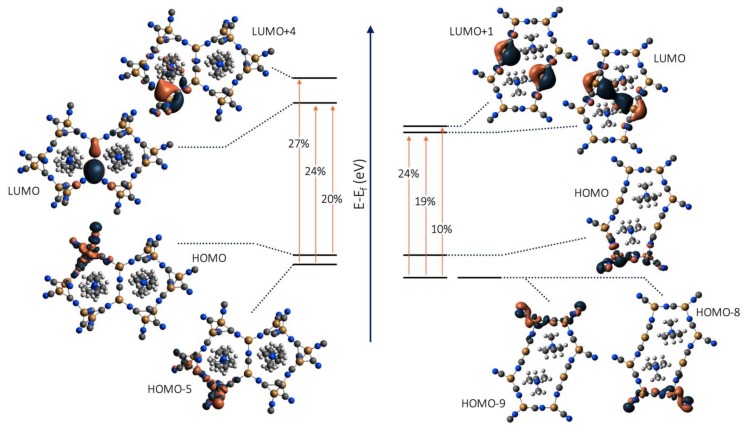
M06/CEP-31G(d) TD-DFT calculated isodensity representations of molecular orbital transitions (≥10% contribution) of (**left**) **1** and (**right**) **2**. Calculated at excited state energies for **1** = 300 nm and **2** = 330 nm. A complete list of negligible (<10% contribution) MO transitions can be found in the Supplementary Materials.

Assignment of the emission band for **2** is the most straightforward given that it displays phosphorescence behavior at all temperatures. Due to the lack of cuprophilic interactions, the triplet emission of **2** is likely the result of either MLCT or mixed metal/ligand to metal charge transfer (M/LMCT) between inequivalent Cu centers. To determine the accurate assignment, we have performed DFT and TD-DFT calculations. TD-DFT calculations of **2** predict an excited state at 330 nm (f-oscillation = 0.0265) in general agreement with the experimental value of 332 nm at 78 K. Molecular orbital calculated isodensity representations, Figure 7, predict that the electron donating MOs are primarily composed of the Cu 3*d* atomic orbitals with contributions from the C/N 2*p*, all residing strictly on the trigonal planar metal centers. Conversely, the electron accepting MOs at this excitation energy are composed of both the Cu 4*s/p* and CN π^*^ orbitals localized to the C_∞v_ group. This calculated transition at 330 nm for **2** clearly indicates that emission from this complex is associated with rearrangement of electrons from the outer D_3h_ CuCN motifs to the inner C_∞v_ CuCN sites. This is consistent with a M/LMCT and agrees with the experimental data as well as reports of similar systems [30,74,75,76]. In a similar fashion, TD-DFT calculations were also performed on a model of **1** to elucidate the nature of the observed dual singlet/triplet emission. At 78 K under 315 nm excitation, **1** displays two dominant bands at 524 nm and 416 nm. Calculations predict a strong transition (f-oscillation = 0.0182) at 300 nm, in general agreement with the observed excitation value of 315 nm. MO transitions at this energy are shown in Figure 7. Unlike the MO transitions predicted for **2**, the nature of the electron accepting MOs for **1** are distinctly different. In all cases the electron donating MOs are the HOMO/HOMO-5, both composed of the {Cu_2_(CN)_4_(*μ*_2_-CN)_2_} rhomboid Cu 3*d* and C/N 2*s* atomic orbitals. However, in one case the electron accepting MO (LUMO+4) is composed of the {Cu_2_(CN)_2_(*μ*_2_-CN)_2_} rhomboid Cu 4*s* and CN π* orbitals while in the other case the electron accepting MO (LUMO) is composed of the trigonal planar Cu 4*s/p* and *μ*^1^-CN π*. These two transitions can best be described as (1) rearrangement of electrons within the {Cu_2_(CN)_4_(*μ*_2_-CN)_2_} rhomboid (^1^MMLCT) and (2) electron transfer from the {Cu_2_(CN)_4_(*μ*_2_-CN)_2_} rhomboid to the trigonal planar {Cu(CN)_3_} (^2^MMLCT). A distinct difference to highlight is the presence of metallophilic interactions involved in the electron accepting MOs of the ^1^MMLCT. Previous reports by us and others have shown that metallophilic interactions shift emission of the d^10^ cyanometallates to lower energies [17,77,78,79,80]. As such, we assign the 542 nm and 416 nm emission bands to a ^1^MMLCT and ^2^MMLCT, respectively.

### 3.4. Optical Memory Behavior

In order to explore the potential use of these systems in optical digital storage devices we have measured the optical memory behavior of **1** and **2**. To perform these experiments we cool the samples to 78 K and irradiate them over a period of time using a 255 nm pulse laser. Emission spectra are measured at various time intervals until no further change in the emission spectra is observed. To restore the emission back to its original intensity we heat the sample to 298 K and then re-cool it to 78 K. Samples that undergo a loss and complete recovery of emission intensity are considered to show optical memory behavior. In our experiments the laser beam focal point (area of irradiation) is less than that of the excitation source, meaning a significant area of the sample surface is left non-irradiated during emission measurements. To compare the rates of emission reduction between **1** and **2**, the emission signal of this non-irradiated area is blanked by subtracting the final irradiation measurement from each of the spectra once no change in emission intensity is observed. Untreated emission spectra of **1** and **2** during irradiation experiments can be found in the Supplementary Materials.

**Figure 8:**
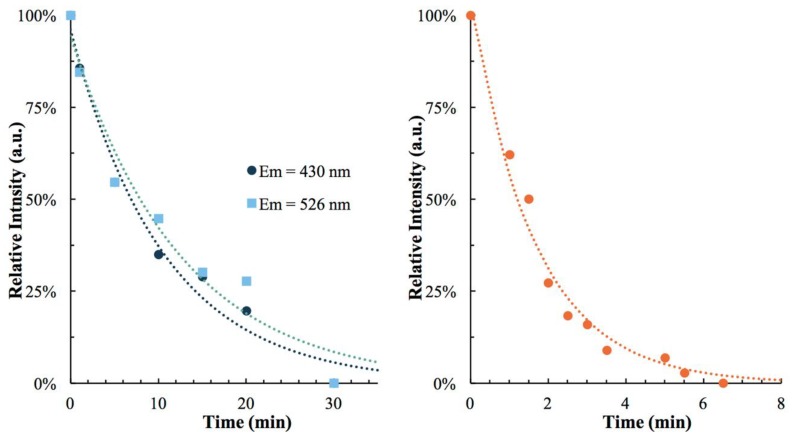
Relative emission loss over time for **1** (**left**) and **2** (**right**) at 78 K under 255 nm laser irradiation. Note that the emission signal of **1** is deconstructed to show the independent intensities of the dominant emission peak at 430 nm and the less intense emission peak at 526 nm.

As seen in Figure 8, both complexes **1** and **2** undergo loss of emission intensity with irradiation. This loss is completely recoverable in both cases by heating to 298 K and recooling to 78 K with quantitative emission intensity recovery of 104% and 101% for **1** and **2**, respectively. We consider these values to be within experimental error of 100%. Complex **2** reaches maximum intensity reduction over a 6.5 min. period, much faster than **1** whose reduction occurs over 30 min.

Variable temperature optical memory experiments were performed on the superior-performing **2** in order to explore the kinetic and activation energy associated with this complex. To investigate the kinetics of the photochemical reaction in **2**, we first consider the elementary reversible reaction:

$$2+h\nu \begin{array}{c} k_{1}\\ ⇄\\ k_{-1} \end{array}2^{*}$$

**Figure 9:**
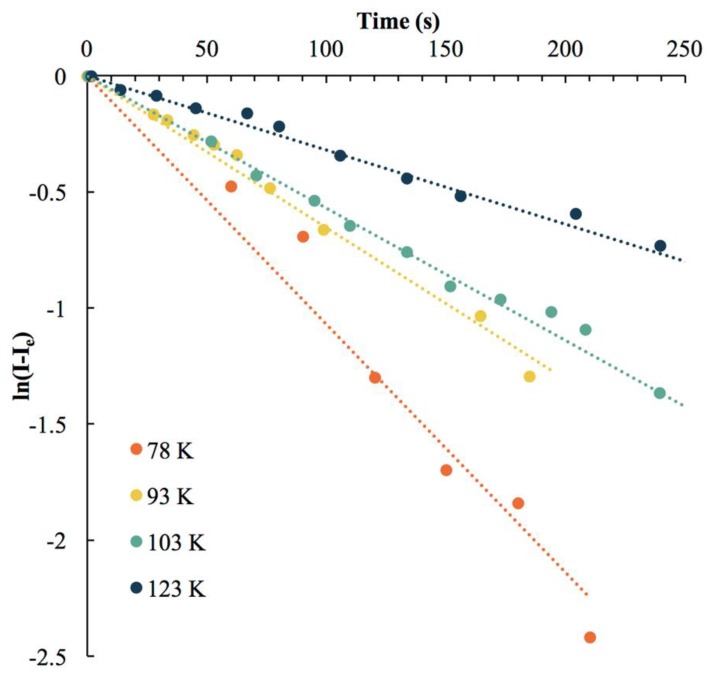
Natural log of emission intensity of **2** versus time after 255 nm laser irradiation at variable temperatures.

In this reaction the luminescence species **2** is photochemically converted to a non-luminescent complex denoted as 2*. The high power input of the laser allows us to assume that the photon supply by the laser is in excess, reducing the elementary equation above via flooding to a simple reversible first order reaction. As discussed elsewhere [81], integration of these simple reactions affords the equation:

$$\ln\{\frac{{\left[I\right]}_{t}-{\left[I\right]}_{\infty }}{{\left[I\right]}_{0}-{\left[I\right]}_{\infty }}\}=-\left(k_{1}+k_{-1}\right)t$$

where I_t_ is the normalized intensity at time t, I_e_ is the intensity at equilibrium, and *k*_1_ and *k*_−1_ are the forward and reverse rate constants, respectively [81]. Thus, a plot of ln{[I]_t_−[I]_e_} versus time should yield a linear line with a slope whose negative is the sum *k*_1_+*k*_−1_, defined as *k*_e_, Figure 9. At low temperatures we are able to maintain the reduced emission intensity without continuous irradiation. Thus, at 78 K the forward rate is much larger than the reverse rate which can be considered zero or negligible. We can then simplify the term *k*_e_ = *k*_1_ + *k*_−1_ to be *k*_e_ = *k*_1_ at low temperatures (for **2** at 78 K *k*_e_ = *k*_1_ = 1.07 × 10^−2^ s^−1^). We expect the forward photochemical reaction is dependent only on the laser energy and is independent of temperature. Instead, it is the reverse reaction that is temperature dependent. So, upon heating the sample, the forward reaction remains constant while the reverse rate increases and becomes non-negligible. Continuous heating eventually results in *k*_−1_ = *k*_1_ which is evident by a decrease of *k*_e_ with increasing temperature and a lack of intensity reduction with laser irradiation. This temperature represents the maximum operational temperature for an optical memory active complex. As expected, increasing the temperature during irradiation at 255 nm for **2** results in a decrease in the emission intensity reduction rate. Since we can now calculate *k*_1_ and *k*_−1_ at different temperatures we can also readily calculate K_eq_ and plot ln(K_eq_) versus temperature to obtain the recovery activation energy via the Arrhenius equation. For **2** we calculate an activation energy of the recovery reaction of 1,886 J/mol for which using the Boltzmann constant equates an operational temperature of 227 K. 

### 3.5. Optical Memory Mechanism

As previously mentioned, our group reported extensively on the optical memory of d^10^ cyanometallates [16,17,23,30,82]. In many of these cases the optical memory behavior is the result of a photo-induced electron transfer [18,24,82]. For example, in the instance of Tl[Ag(CN)_2_] irradiation at 78 K using a 318 nm laser results in the formation of a redox active [Ag(CN)_2_]_3_^3−^ trimer. This exciplex monomer readily accepts an electron from Tl^+^. Through a cascading redox reaction, the Tl^+^ is oxidized to Tl^+3^ while the [Ag(CN)_2_]_3_^3−^ trimer is photochemically reduced to [Ag(CN)_2_]_3_^4−^. In this reaction the reduced [Ag(CN)_2_]_3_^4−^ trimer is not luminescent and, thus, responsible for the loss of emission over time. This reaction is completely reversible upon heating the sample to room temperature. 

Given the precedent for M(CN)_2_^−^-based systems to display photo-induced electron transfer optical memory, we propose a similar mechanism to explain optical memory in **1** and **2**. The cation Me_4_N^+^ is not redox active, nor does it strongly interact with the Cu(CN)_2_^−^-based anions of **1** and **2**. As such, it is unlikely that the Me_4_N^+^ cation plays a role in optical memory behavior. This leads us to consider only the Cu(CN)_2_^−^-based units as the active emissive species in a series of redox reactions. We use the standard reduction potentials of Cu and Cu(CN)_2_^−^ complexes to describe these reactions [83,84]. Photo-oxidation of Cu(I) in Cu(CN)_2_^−^ to form Cu(II) according to Reaction (1) is a non-spontaneous equation with a standard reduction potential of −1.103 V (1.103 eV):

(1) Cu(CN)_2_^−^ + Cu(CN)_2_^−^ + hⱱ ➝ 2Cu^2+^ + 4CN^−^ + 2e^−^  E^o^ = −1.103 V

Irradiation using a 255 nm (4.86 eV) laser provides ample energy to overcome the reaction’s energetic penalty. It is generally understood that Cu^2+^ acts as a MLCT-quenching species. Thus, we believe Cu^2+^ acts as the non-luminescent species during laser irradiation. In Reaction (2) we recombine the electrons produced in Reaction (1) to reduce a Cu^2+^ center to Cu(0). This reaction is spontaneous in the forward direction, as evidenced by the reduction potential (*E*^o^ = 0.339 V). Importantly, the combination of Cu^2+^ and Cu(0), Reaction (3), is non-spontaneous:

(2) Cu^2+^ + 2e^−^ ➝ Cu  E^o^ = 0.339 V(3) Cu^2+^ + Cu ➝ 2Cu^+^  E^o^ = −0.357 V

The non-spontaneity of Reaction (3) in the forward direction and of reaction (2) in the reverse direction produces a metastable state wherein the non-luminescent photochemical product is unable to return back to its original state. To return to the original state we propose oxidation of Cu by the free cyano groups to produce an electron, Reaction (4). While Reaction (4) is spontaneous, by keeping the solid at low temperatures it is kinetically unable to proceed. Heating to 298 K provides ample energy to the system to allow oxidation of Cu into Cu^+^. The resulting electron is then transferred to the remaining Cu^2+^ to form Cu^+^ (*E*^o^ = 0.161 V), which undergoes coordination of two cyano units, Reactions (5)/(6), completing the recovery back to the original state.

(4) Cu + 2CN^−^ ➝ Cu(CN)_2_^−^ + e^−^  E^o^ = 0.429 V(5) Cu^2+^ + e^−^ ➝ Cu^+^  E^o^ = 0.161 V(6) Cu^+^ + 2CN^−^ ➝ Cu(CN)_2_^−^  E^o^ = 0 V

The proposed mechanism offers an explanation for the difference in optical memory behavior between **1** and **2** with respect to the emission intensity reduction rates. In **1**, due to metallophilic interactions, both triplet and singlet emission bands are observed while for compound **2** only triplet emission is observed. Because both species share a triplet emission pathway and both undergo photo-induced emission intensity reduction, it seems likely that the triplet pathway is responsible for optical memory behavior. This conclusion is supported by the observed slower emission reduction rate of **1** whose short-lived singlet emission pathway could act competitively against the photo-induced electron transfer mechanism.

## 4. Conclusions

We have reported on three CuCN networks **1**, **2**, and **3**. While all of the complexes are synthesized from aqueous solutions of KCN/CuCN and Me_4_NCl, they display distinctly different structural and photophysical behavior. In the case of **1**, a 3D network of {Cu_2_(CN)_4_(*μ*_2_-CN)_2_} rhomboids crosslinked by cyano ligands and D_3h_ {Cu(CN)_3_} metal clusters is formed. Metallophilic interactions are observed between rhomboid Cu centers having short Cu⋯Cu distances. Complex **2** consists of layered 2D sheets formed by elongated fused hexagonal {Cu_8_(CN)_8_} rings. Metallophilic interactions are notably absent. Complex **3**, which could not be reproducibly synthesized, was a non-emissive, simple hexagonal 2D network. Complex **1** emits bright green at 298 K and blue at 78 K while **2** emits bright blue at all temperatures. Time-dependent luminescence measurements reveal that **1** is a dual singlet/triplet emitter while **2** is strictly triplet in nature. Both complexes are optical memory active, with **2** showing significantly greater emission intensity reduction. Kinetic studies show this complex has a recovery activation energy of 1,866 J/mol or 227 K. We have proposed a light-induced electron transfer as the mechanism by which emission intensity is lost and recovered. Our findings show that triplet emission is responsible for optical memory activity in these complexes and that the singlet emission in **1** actively competes with the optical memory active triplet pathway. Our findings indicate that complexes which exhibit singlet emission are poor candidates for optical memory complexes that proceed through a photo-induced electron transfer.

## Figures and Tables

**Table 1:** Crystal and structure refinement data. ^a.^

Complex	1	2		3
CCDC deposit number	1903299	1903300	1903302	1903301
color and habit	Colorless needle	Colorless needle	Colorless needle	Colorless block
Size, mm	0.130 × 0.040 × 0.020	0.270 × 0.020 × 0.020	0.310 × 0.020 × 0.020	0.290 × 0.150 × 0.110
formula	C_14_H_24_Cu_4_N_8_O_0.25_	C_8_H_12_Cu_3_N_5_	C_16_H_24_Cu_6_N_10_	C_7_H_12_Cu_2_N_4_
formula weight	563.07	368.85	737.69	279.29
space group	*Pnma*	*C2/c*	*P-1*	*P-1*
a, Å	16.1310(4)	15.1615(2)	8.7275(2)	8.501(3)
b, Å	13.1438(3)	8.57220(10)	10.5197(2)	8.751(3)
c, Å	19.6376(5)	12.43490(10)	14.8175(4)	9.047(3)
α, °	90	90	69.2830(10)	78.376(6)
β, °	90	127.2540(10)	80.262(2)	63.283(5)
γ, °	90	90	78.8820(10)	61.151(5)
volume, Å^3^	4163.62(18)	1286.38(3)	1240.99(5)	526.5(3)
*Z*	2	4	2	2
ρ_calc_, Mg cm^−3^	1.797	1.905	1.974	1.762
F_000_	2260	728	728	280
*μ*, mm^−1^	4.729	5.598	5.802	3.999
Radiation	Cu Kα (1.54178 Å)	Cu Kα (1.54178 Å)	Cu Kα (1.54178 Å)	Mo Kα (0.71073 Å)
Temp., K	100	296	100	100
residuals: ^a^ R; R_w_	0.0439, 0.1216	0.0267, 0.0763	0.0356, 0.0849	0.0168, 0.0428
goodness of fit	1.078	1.084	1.101	1.062

^a^ R = *R*_1_ = ∑|*F_o_*| − |*F_c_*||/∑|*F_o_*| for observed data only. R_w_ = *wR*_2_ = {∑ [*w*(*F_o_*^2^ − *F_c_*^2^)^2^]/∑[*w*(*F_o_*^2^)^2^]}^1/2^ for all data.

**Table 2:** Summary of luminescence spectra of **1** and **2** between 78 K and 298 K.

Complex	Temperature	Ex *λ*_max_	Em *λ*_max_	Lifetime
**1**	298 K	334 nm	524 nm	63 *μ*s
	78 K	315 nm	416 nm	78 ns
			542 nm	0.31 *μ*s
**2**	298 K	340 nm	420 nm	6.3 *μ*s
	78 K	332 nm	440 nm	33 *μ*s

## Supplementary Materials

Supplementary materials are available online at https://www.mdpi.com/1996-1944/12/8/1211/s1. Figure S1: Unit cell diagram of 1 viewed along the b-axis, Figure S2: Unit cell diagram of 2 at 298 K viewed between the a- and c-axes, Figure S3: Unit cell diagram of 2 at 100 K viewed along the a-axis, Figure S4: Unit cell diagram of 3 at 100 K viewed along the b-axis, Figure S5: Vibrational infrared spectra of solid samples of 1 and 2 at 298 K, Figure S6: Luminescence spectrum of (left) 1 and (right) 2 at 78 K and 298 K, Figure S7: Lifetime measurements of 1 at (left) 298 K (*λ*em = 522 nm) and (right) 78 K, Figure S8: Lifetime measurements of 2 at (left) 298 K (*λ*em = 420 nm) and (right) 78 K (*λ*em = 440 nm), Figure S9: M06/CEP-31G(d) ground state structure of 1 (left) and 2 (right), Figure S10: Uncorrected optical memory results of (left) 1 and (right) 2, Table S1: Select bond lengths of 1, Table S2: Selected bond angles of 1, Table S3: Select bond length of 2 at 296 K, Table S4: Selected bond angles of 2 at 296 K, Table S5: Selected bond lengths of 2 at 100 K, Table S6: Selected bond angles of 2 at 100 K, Table S7: DFT ground state parameters of 1 compared to experimental values, Table S8: DFT ground state parameters of 2 compared to experimental values, Table S9: M06/CEP-31G(d) TD-DFT calculation excited state transitions for 1 at 300 nm, Table S10: M06/CEP-31G(d) TD-DFT calculation excited state transitions for 2 at 330 nm.
