# Supplementary material for: Synthesis and Luminescence of Optical Memory Active Tetramethylammonium Cyanocuprate(I) 3D Networks
Source: Materials (Basel). 2019 Apr 12;12(8):1211. doi: 10.3390/ma12081211 (PMC6514951; doi:10.3390/ma12081211)
Supplement: Supplementary file 1 [file materials-12-01211-s001.pdf]

materials

Supplementary Materials

Synthesis and Luminescence of Optical Memory Active Tetramethylammonium Cyanocuprate(I) 3D Networks

Aaron D. Nicholas [1,*], Rebeka M. Bullard [1], Amelia M. Wheaton [2], Michaela Streep [2], Victoria A. Nicholas [1], Robert D. Pike [2] and Howard H. Patterson [1]

[1] Department of Chemistry, University of Maine, Orono, ME 04469, USA; rebeka.bullard@maine.edu (R.M.B.); victoria.nicholas@maine.edu (V.A.N.); howardp@maine.edu (H.H.P.)
[2] Department of Chemistry, College of William and Mary, Williamsburg, VA 23187-8795, USA; amwheaton@wisc.edu (A.M.W.); mestreep@email.wm.edu (M.S); rdpike@wm.edu (R.D.P.)
* Correspondence: aaron.d.nicholas@maine.edu; Tel.: +1-207-581-1178

Received: 24 March 2019; Accepted: 10 April 2019; Published: date

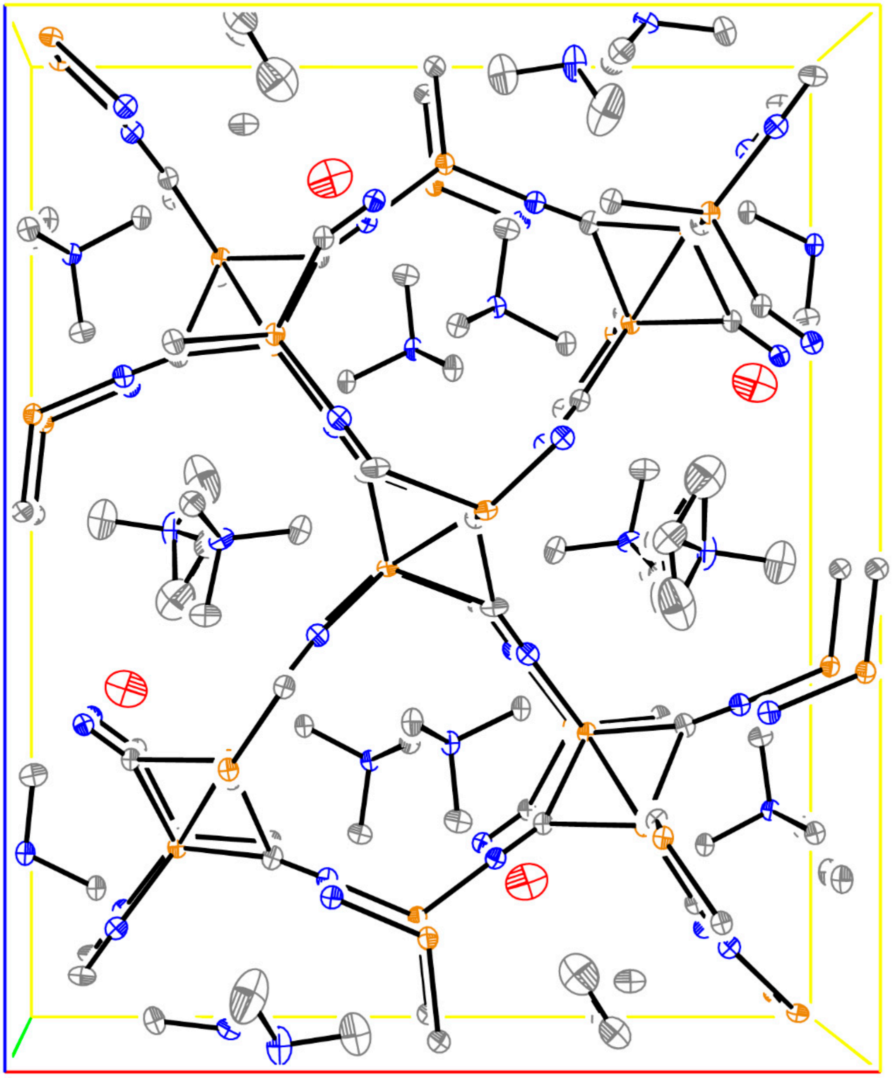

Figure S1. Unit cell diagram of **1** viewed along the *b*-axis, 30% ellipsoids. Disordered atom positions and hydrogen atoms omitted.

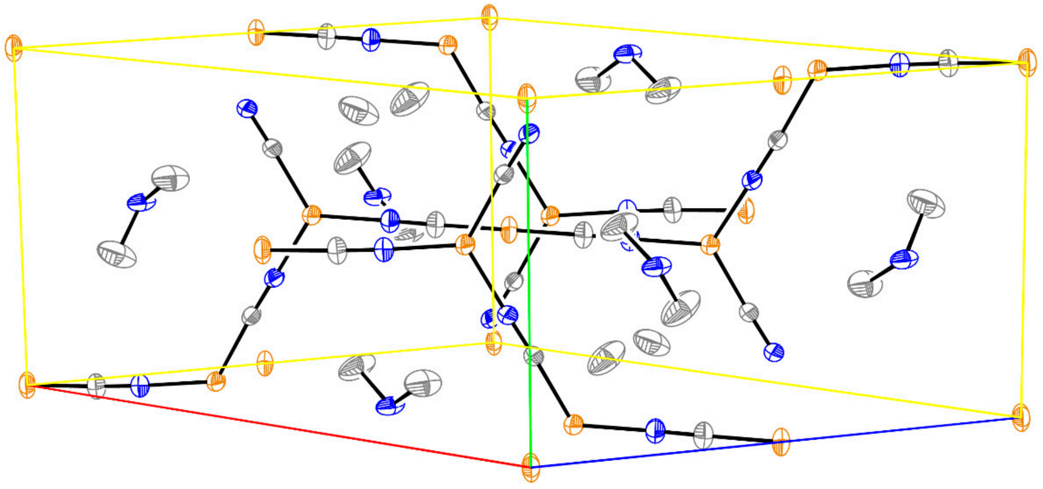

Figure S2. Unit cell diagram of **2** at 296 K viewed between the *a*- and *c*-axes, 30% ellipsoids. Disordered atom positions and hydrogen atoms omitted.

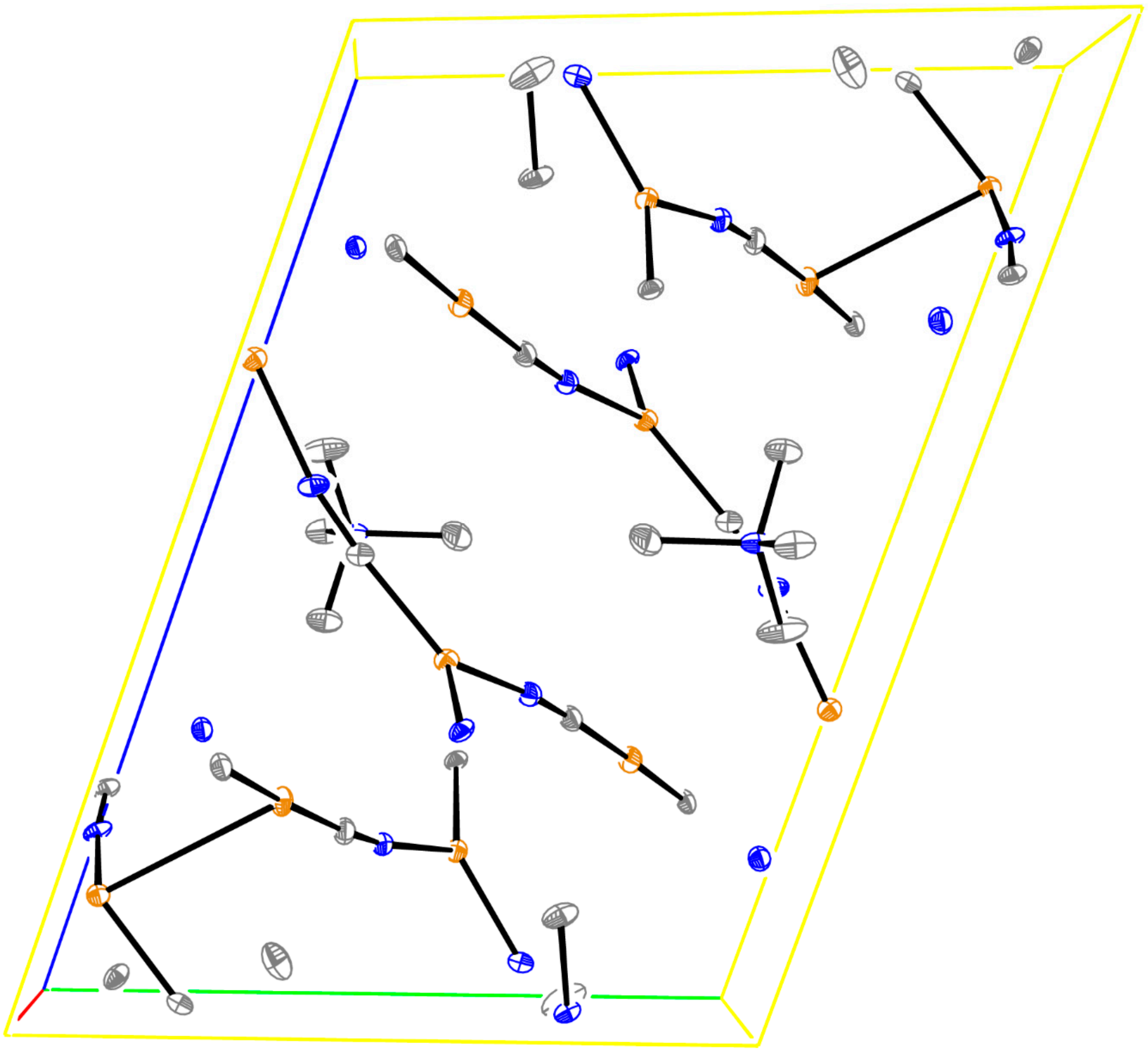

Figure S3. Unit cell diagram of **2** at 100 K viewed along the *a*-axis, 30% ellipsoids. Disordered atom positions and hydrogen atoms omitted.

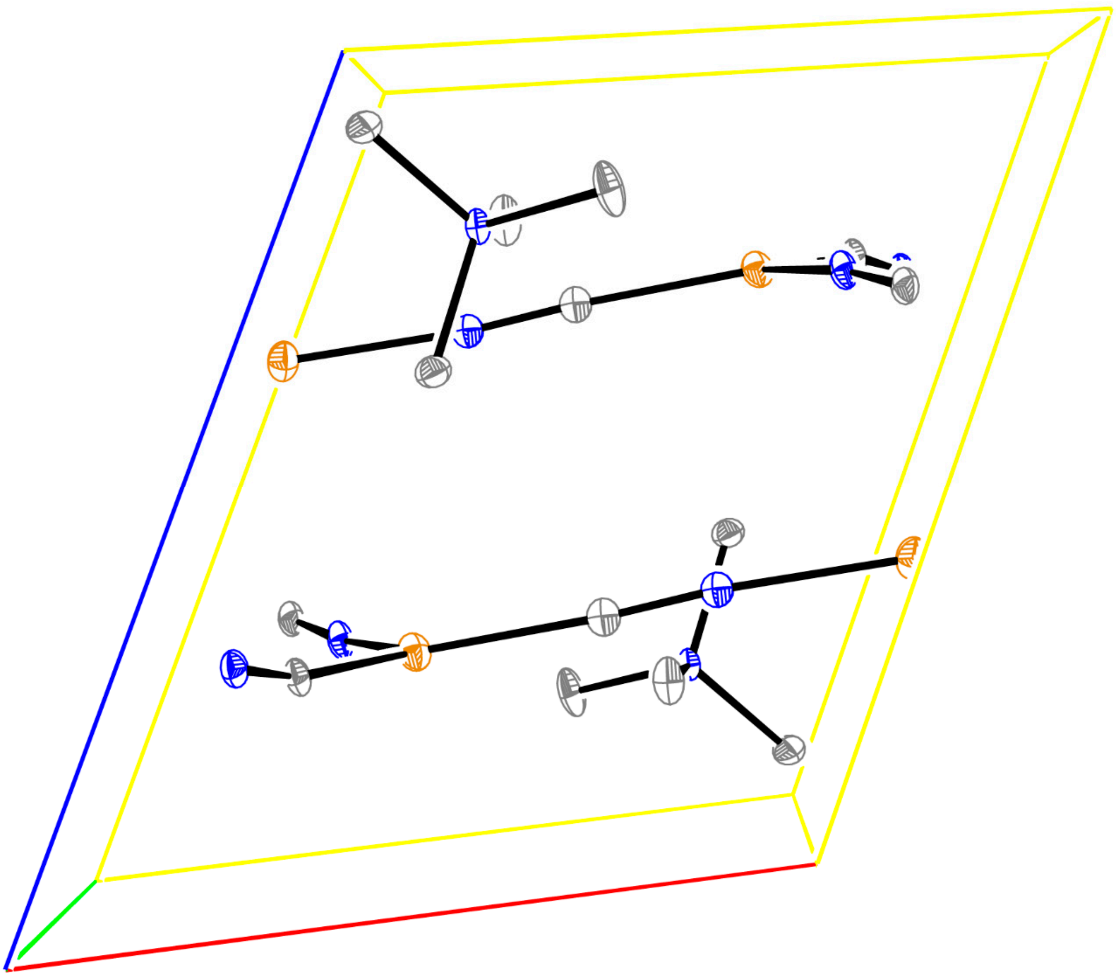

Figure S4. Unit cell diagram of **3** at 100 K viewed along the *b*-axis, 30% ellipsoids. Disordered atom positions and hydrogen atoms omitted.

Table S1. Select bond lengths of **1**.

Cu(1)-N(1B)	1.960(4)	C(10)-H(10A)	0.9800
Cu(1)-C(1A)	1.960(4)	C(10)-H(10B)	0.9800
Cu(1)-N(8)	1.963(4)	C(10)-H(10C)	0.9800
Cu(1)-C(8)	1.963(4)	C(11)-H(11A)	0.9800
Cu(1)-C(4)#1	2.089(4)	C(11)-H(11B)	0.9800
Cu(1)-C(3)#2	2.236(4)	C(11)-H(11C)	0.9800
Cu(1)-Cu(3)#2	2.5146(8)	N(10)-C(14)	1.487(8)
Cu(3)-N(7)	1.987(4)	N(10)-C(13)#8	1.492(5)
Cu(3)-C(7)	1.987(4)	N(10)-C(13)	1.492(5)
Cu(3)-C(3)	2.007(4)	N(10)-C(12)	1.497(7)
Cu(3)-N(2)	2.023(4)	C(12)-H(12A)	0.9800
Cu(3)-C(4)#3	2.077(4)	C(12)-H(12B)	0.9800
Cu(3)-Cu(1)#4	2.5146(8)	C(12)-H(12C)	0.9800
Cu(4)-N(5)	1.905(5)	C(13)-H(13A)	0.9800
Cu(4)-C(5)	1.905(5)	C(13)-H(13B)	0.9800
Cu(4)-N(4)	1.913(4)	C(13)-H(13C)	0.9800
Cu(4)-N(3)	1.923(4)	C(14)-H(14A)	0.9800
N(2)-C(2)	1.145(6)	C(14)-H(14B)	0.9800

C(2)-Cu(2B)	1.934(15)	C(14)-H(14C)	0.9800
C(2)-Cu(2A)	2.037(6)	N(11)-C(16)	1.433(11)
C(2)-Cu(2A)#5	2.299(9)	N(11)-C(18)	1.451(16)
N(3)-C(3)	1.151(6)	N(11)-C(15)	1.458(12)
C(3)-Cu(1)#4	2.236(4)	N(11)-C(17)	1.595(17)
N(4)-C(4)	1.152(6)	C(15)-H(15A)	0.9800
C(4)-Cu(3)#6	2.077(4)	C(15)-H(15B)	0.9800
C(4)-Cu(1)#1	2.089(4)	C(15)-H(15C)	0.9800
Cu(2A)-C(6)	1.981(6)	C(16)-H(16A)	0.9800
Cu(2A)-N(1A)	2.005(6)	C(16)-H(16B)	0.9800
Cu(2A)-C(2)#5	2.299(9)	C(16)-H(16C)	0.9800
Cu(2A)-Cu(2A)#5	2.436(12)	C(17)-H(17A)	0.9800
N(1A)-C(1A)	1.161(6)	C(17)-H(17B)	0.9800
C(5)-C(5)#1	1.137(10)	C(17)-H(17C)	0.9800
C(6)-C(6)#7	1.180(8)	C(18)-H(18A)	0.9800
C(7)-C(7)#8	1.167(8)	C(18)-H(18B)	0.9800
C(8)-C(8)#8	1.151(8)	C(18)-H(18C)	0.9800
Cu(2B)-N(6)	1.910(11)	N(12)-C(21)	1.475(9)
Cu(2B)-C(1B)	1.912(13)	N(12)-C(20)#8	1.491(5)
Cu(2B)-Cu(2B)#5	3.03(8)	N(12)-C(20)	1.491(5)
C(1B)-N(1B)	1.161(6)	N(12)-C(19)	1.498(9)
N(5)-N(5)#1	1.137(10)	C(19)-H(19A)	0.9800
N(6)-N(6)#7	1.180(8)	C(19)-H(19B)	0.9800
N(7)-N(7)#8	1.167(8)	C(19)-H(19C)	0.9800
N(8)-N(8)#8	1.151(8)	C(20)-H(20A)	0.9800
N(9)-C(10)	1.489(8)	C(20)-H(20B)	0.9800
N(9)-C(9)	1.496(8)	C(20)-H(20C)	0.9800
N(9)-C(11)#7	1.499(5)	C(21)-H(21A)	0.9800
N(9)-C(11)	1.499(5)	C(21)-H(21B)	0.9800
C(9)-H(9A)	0.9800	C(21)-H(21C)	0.9800
C(9)-H(9B)	0.9800	O(1)-H(1W)	0.82(2)
C(9)-H(9C)	0.9800	O(1)-H(2W)	0.82(2)

Table S2. Selected bond angles of **1**.

N(1B)-Cu(1)-N(8)	114.59(15)	N(9)-C(10)-H(10B)	109.5
C(1A)-Cu(1)-C(8)	114.59(15)	H(10A)-C(10)-H(10B)	109.5
N(1B)-Cu(1)-C(4)#1	111.26(16)	N(9)-C(10)-H(10C)	109.5
C(1A)-Cu(1)-C(4)#1	111.26(16)	H(10A)-C(10)-H(10C)	109.5
N(8)-Cu(1)-C(4)#1	113.11(15)	H(10B)-C(10)-H(10C)	109.5
C(8)-Cu(1)-C(4)#1	113.11(15)	N(9)-C(11)-H(11A)	109.5
N(1B)-Cu(1)-C(3)#2	111.54(16)	N(9)-C(11)-H(11B)	109.5
C(1A)-Cu(1)-C(3)#2	111.54(16)	H(11A)-C(11)-H(11B)	109.5
N(8)-Cu(1)-C(3)#2	103.14(16)	N(9)-C(11)-H(11C)	109.5
C(8)-Cu(1)-C(3)#2	103.14(16)	H(11A)-C(11)-H(11C)	109.5
C(4)#1-Cu(1)-C(3)#2	102.21(16)	H(11B)-C(11)-H(11C)	109.5
N(1B)-Cu(1)-Cu(3)#2	124.97(11)	C(14)-N(10)-C(13)#8	109.7(3)
C(1A)-Cu(1)-Cu(3)#2	124.97(11)	C(14)-N(10)-C(13)	109.7(3)
N(8)-Cu(1)-Cu(3)#2	119.91(11)	C(13)#8-N(10)-C(13)	109.3(5)
C(8)-Cu(1)-Cu(3)#2	119.91(11)	C(14)-N(10)-C(12)	110.2(5)
C(4)#1-Cu(1)-Cu(3)#2	52.66(11)	C(13)#8-N(10)-C(12)	108.9(3)
C(3)#2-Cu(1)-Cu(3)#2	49.56(11)	C(13)-N(10)-C(12)	108.9(3)
N(7)-Cu(3)-C(3)	110.75(16)	N(10)-C(12)-H(12A)	109.5
C(7)-Cu(3)-C(3)	110.75(16)	N(10)-C(12)-H(12B)	109.5
N(7)-Cu(3)-N(2)	105.75(15)	H(12A)-C(12)-H(12B)	109.5
C(7)-Cu(3)-N(2)	105.75(15)	N(10)-C(12)-H(12C)	109.5
C(3)-Cu(3)-N(2)	113.45(17)	H(12A)-C(12)-H(12C)	109.5
N(7)-Cu(3)-C(4)#3	106.79(16)	H(12B)-C(12)-H(12C)	109.5
C(7)-Cu(3)-C(4)#3	106.79(16)	N(10)-C(13)-H(13A)	109.5

C(3)-Cu(3)-C(4)#3	111.07(17)	N(10)-C(13)-H(13B)	109.5
N(2)-Cu(3)-C(4)#3	108.69(15)	H(13A)-C(13)-H(13B)	109.5
N(7)-Cu(3)-Cu(1)#4	123.84(11)	N(10)-C(13)-H(13C)	109.5
C(7)-Cu(3)-Cu(1)#4	123.84(11)	H(13A)-C(13)-H(13C)	109.5
C(3)-Cu(3)-Cu(1)#4	57.98(13)	H(13B)-C(13)-H(13C)	109.5
N(2)-Cu(3)-Cu(1)#4	129.85(10)	N(10)-C(14)-H(14A)	109.5
C(4)#3-Cu(3)-Cu(1)#4	53.09(11)	N(10)-C(14)-H(14B)	109.5
N(5)-Cu(4)-N(4)	120.58(18)	H(14A)-C(14)-H(14B)	109.5
C(5)-Cu(4)-N(4)	120.58(18)	N(10)-C(14)-H(14C)	109.5
N(5)-Cu(4)-N(3)	119.12(18)	H(14A)-C(14)-H(14C)	109.5
C(5)-Cu(4)-N(3)	119.12(18)	H(14B)-C(14)-H(14C)	109.5
N(4)-Cu(4)-N(3)	120.28(15)	C(16)-N(11)-C(18)	114.8(10)
C(2)-N(2)-Cu(3)	174.4(3)	C(16)-N(11)-C(15)	122.3(6)
N(2)-C(2)-Cu(2B)	141.9(15)	C(18)-N(11)-C(15)	106.3(10)
N(2)-C(2)-Cu(2A)	153.2(5)	C(16)-N(11)-C(17)	105.5(9)
N(2)-C(2)-Cu(2A)#5	138.7(4)	C(18)-N(11)-C(17)	106.5(12)
Cu(2A)-C(2)-Cu(2A)#5	68.1(3)	C(15)-N(11)-C(17)	99.3(9)
C(3)-N(3)-Cu(4)	171.4(4)	N(11)-C(15)-H(15A)	109.5
N(3)-C(3)-Cu(3)	158.9(4)	N(11)-C(15)-H(15B)	109.5
N(3)-C(3)-Cu(1)#4	128.2(4)	H(15A)-C(15)-H(15B)	109.5
Cu(3)-C(3)-Cu(1)#4	72.46(14)	N(11)-C(15)-H(15C)	109.5
C(4)-N(4)-Cu(4)	177.7(4)	H(15A)-C(15)-H(15C)	109.5
N(4)-C(4)-Cu(3)#6	148.0(3)	H(15B)-C(15)-H(15C)	109.5
N(4)-C(4)-Cu(1)#1	137.7(3)	N(11)-C(16)-H(16A)	109.5
Cu(3)#6-C(4)-Cu(1)#1	74.25(14)	N(11)-C(16)-H(16B)	109.5
C(6)-Cu(2A)-N(1A)	111.0(3)	H(16A)-C(16)-H(16B)	109.5
C(6)-Cu(2A)-C(2)	110.1(3)	N(11)-C(16)-H(16C)	109.5
N(1A)-Cu(2A)-C(2)	110.4(3)	H(16A)-C(16)-H(16C)	109.5
C(6)-Cu(2A)-C(2)#5	104.6(3)	H(16B)-C(16)-H(16C)	109.5
N(1A)-Cu(2A)-C(2)#5	108.6(3)	N(11)-C(17)-H(17A)	109.5
C(2)-Cu(2A)-C(2)#5	111.9(3)	N(11)-C(17)-H(17B)	109.5
C(6)-Cu(2A)-Cu(2A)#5	121.7(3)	H(17A)-C(17)-H(17B)	109.5
N(1A)-Cu(2A)-Cu(2A)#5	126.4(4)	N(11)-C(17)-H(17C)	109.5
C(2)-Cu(2A)-Cu(2A)#5	61.1(2)	H(17A)-C(17)-H(17C)	109.5
C(2)#5-Cu(2A)-Cu(2A)#5	50.9(3)	H(17B)-C(17)-H(17C)	109.5
C(1A)-N(1A)-Cu(2A)	172.1(4)	N(11)-C(18)-H(18A)	109.5
N(1A)-C(1A)-Cu(1)	174.1(3)	N(11)-C(18)-H(18B)	109.5
C(5)#1-C(5)-Cu(4)	179.2(7)	H(18A)-C(18)-H(18B)	109.5
C(6)#7-C(6)-Cu(2A)	175.12(18)	N(11)-C(18)-H(18C)	109.5
C(7)#8-C(7)-Cu(3)	177.58(11)	H(18A)-C(18)-H(18C)	109.5
C(8)#8-C(8)-Cu(1)	172.67(11)	H(18B)-C(18)-H(18C)	109.5
N(6)-Cu(2B)-C(1B)	118.5(7)	C(21)-N(12)-C(20)#8	110.7(4)
N(6)-Cu(2B)-C(2)	117.9(7)	C(21)-N(12)-C(20)	110.7(4)
C(1B)-Cu(2B)-C(2)	119.4(10)	C(20)#8-N(12)-C(20)	110.4(5)
N(6)-Cu(2B)-Cu(2B)#5	110.2(18)	C(21)-N(12)-C(19)	108.7(5)
C(1B)-Cu(2B)-Cu(2B)#5	115.1(16)	C(20)#8-N(12)-C(19)	108.1(3)
C(2)-Cu(2B)-Cu(2B)#5	61.5(5)	C(20)-N(12)-C(19)	108.1(4)
N(1B)-C(1B)-Cu(2B)	173.2(11)	N(12)-C(19)-H(19A)	109.5
C(1B)-N(1B)-Cu(1)	174.1(3)	N(12)-C(19)-H(19B)	109.5
N(5)#1-N(5)-Cu(4)	179.2(7)	H(19A)-C(19)-H(19B)	109.5
N(6)#7-N(6)-Cu(2B)	165.6(14)	N(12)-C(19)-H(19C)	109.5
N(7)#8-N(7)-Cu(3)	177.58(11)	H(19A)-C(19)-H(19C)	109.5
N(8)#8-N(8)-Cu(1)	172.67(11)	H(19B)-C(19)-H(19C)	109.5
C(10)-N(9)-C(9)	109.9(5)	N(12)-C(20)-H(20A)	109.5
C(10)-N(9)-C(11)#7	109.7(3)	N(12)-C(20)-H(20B)	109.5
C(9)-N(9)-C(11)#7	109.2(3)	H(20A)-C(20)-H(20B)	109.5
C(10)-N(9)-C(11)	109.7(3)	N(12)-C(20)-H(20C)	109.5
C(9)-N(9)-C(11)	109.2(3)	H(20A)-C(20)-H(20C)	109.5
C(11)#7-N(9)-C(11)	109.2(4)	H(20B)-C(20)-H(20C)	109.5

N(9)-C(9)-H(9A)	109.5	N(12)-C(21)-H(21A)	109.5
N(9)-C(9)-H(9B)	109.5	N(12)-C(21)-H(21B)	109.5
H(9A)-C(9)-H(9B)	109.5	H(21A)-C(21)-H(21B)	109.5
N(9)-C(9)-H(9C)	109.5	N(12)-C(21)-H(21C)	109.5
H(9A)-C(9)-H(9C)	109.5	H(21A)-C(21)-H(21C)	109.5
H(9B)-C(9)-H(9C)	109.5	H(21B)-C(21)-H(21C)	109.5
N(9)-C(10)-H(10A)	109.5	H(1W)-O(1)-H(2W)	104(3)

Table S3. Select bond length of **2** at 296 K.

Cu(1)-N(1B)	1.917(2)	N(3A)-C(4A)#4	1.522(6)
Cu(1)-C(1A)	1.917(2)	N(3A)-C(4A)	1.522(6)
Cu(1)-C(2B)	1.934(3)	C(3A)-H(3AA)	0.9600
Cu(1)-N(2A)	1.934(3)	C(3A)-H(3AB)	0.9600
Cu(1)-C(1B)#1	1.939(2)	C(3A)-H(3AC)	0.9600
Cu(1)-N(1A)#1	1.939(2)	C(4A)-C(4A)#4	1.957(16)
Cu(2)-N(2B)	1.850(3)	C(4A)-H(4AA)	0.9600
Cu(2)-C(2A)	1.850(3)	C(4A)-H(4AB)	0.9600
Cu(2)-N(2B)#2	1.850(3)	C(4A)-H(4AC)	0.9600
Cu(2)-C(2A)#2	1.850(3)	N(3B)-C(4B)#4	1.384(10)
N(1A)-C(1A)	1.150(3)	N(3B)-C(4B)	1.384(11)
N(1A)-Cu(1)#3	1.939(2)	N(3B)-C(3B)	1.793(12)
C(2A)-N(2A)	1.144(5)	N(3B)-C(3B)#4	1.793(12)
C(1B)-N(1B)	1.150(3)	C(3B)-H(3BA)	0.9600
C(1B)-Cu(1)#3	1.939(2)	C(3B)-H(3BB)	0.9600
N(2B)-C(2B)	1.144(5)	C(3B)-H(3BC)	0.9600
N(3A)-C(3A)#4	1.413(5)	C(4B)-H(4BA)	0.9600
N(3A)-C(3A)	1.413(5)	C(4B)-H(4BB)	0.9600
		C(4B)-H(4BC)	0.9600

Table S4. Selected bond angles of **2** at 298 K.

N(1B)-Cu(1)-C(2B)	122.94(10)	N(3A)-C(4A)-C(4A)#4	50.0(4)
C(1A)-Cu(1)-N(2A)	122.94(10)	N(3A)-C(4A)-H(4AA)	109.5
N(1B)-Cu(1)-C(1B)#1	119.21(9)	C(4A)#4-C(4A)-H(4AA)	75.7
C(2B)-Cu(1)-C(1B)#1	117.53(9)	N(3A)-C(4A)-H(4AB)	109.5
C(1A)-Cu(1)-N(1A)#1	119.21(9)	C(4A)#4-C(4A)-H(4AB)	87.9
N(2A)-Cu(1)-N(1A)#1	117.53(9)	H(4AA)-C(4A)-H(4AB)	109.5
N(2B)-Cu(2)-N(2B)#2	180	N(3A)-C(4A)-H(4AC)	109.5
C(2A)-Cu(2)-C(2A)#2	180	C(4A)#4-C(4A)-H(4AC)	157.8
C(1A)-N(1A)-Cu(1)#3	174.7(3)	H(4AA)-C(4A)-H(4AC)	109.5
N(1A)-C(1A)-Cu(1)	175.0(2)	H(4AB)-C(4A)-H(4AC)	109.5
N(2A)-C(2A)-Cu(2)	177.7(3)	C(4B)#4-N(3B)-C(4B)	176.9(14)
C(2A)-N(2A)-Cu(1)	173.0(3)	C(4B)#4-N(3B)-C(3B)	97.7(7)
N(1B)-C(1B)-Cu(1)#3	174.7(3)	C(4B)-N(3B)-C(3B)	83.6(7)
C(1B)-N(1B)-Cu(1)	175.0(2)	C(4B)#4-N(3B)-C(3B)#4	83.6(7)
C(2B)-N(2B)-Cu(2)	177.7(3)	C(4B)-N(3B)-C(3B)#4	97.7(7)
N(2B)-C(2B)-Cu(1)	173.0(3)	C(3B)-N(3B)-C(3B)#4	128.7(14)
C(3A)#4-N(3A)-C(3A)	114.1(6)	N(3B)-C(3B)-H(3BA)	109.5
C(3A)#4-N(3A)-C(4A)#4	120.1(5)	N(3B)-C(3B)-H(3BB)	109.5
C(3A)-N(3A)-C(4A)#4	109.4(4)	H(3BA)-C(3B)-H(3BB)	109.5
C(3A)#4-N(3A)-C(4A)	109.4(4)	N(3B)-C(3B)-H(3BC)	109.5
C(3A)-N(3A)-C(4A)	120.1(5)	H(3BA)-C(3B)-H(3BC)	109.5
C(4A)#4-N(3A)-C(4A)	80.1(7)	H(3BB)-C(3B)-H(3BC)	109.5
N(3A)-C(3A)-H(3AA)	109.5	N(3B)-C(4B)-H(4BA)	109.5
N(3A)-C(3A)-H(3AB)	109.5	N(3B)-C(4B)-H(4BB)	109.5
H(3AA)-C(3A)-H(3AB)	109.5	H(4BA)-C(4B)-H(4BB)	109.5
N(3A)-C(3A)-H(3AC)	109.5	N(3B)-C(4B)-H(4BC)	109.5
H(3AA)-C(3A)-H(3AC)	109.5	H(4BA)-C(4B)-H(4BC)	109.5
H(3AB)-C(3A)-H(3AC)	109.5	H(4BB)-C(4B)-H(4BC)	109.5

Table S5. Selected bond lengths of **2** at 100 K.

Cu(1A)-C(1A)	1.830(5)	C(8B)-Cu(3)#5	1.944(4)
Cu(1A)-C(2A)	1.877(5)	Cu(2)-Cu(4B)#1	3.015(10)
Cu(1A)-Cu(5)#1	2.913(4)	Cu(2)-Cu(4A)#1	3.059(3)
Cu(4A)-C(5A)	1.821(5)	Cu(3)-C(8B)#5	1.944(4)
Cu(4A)-C(6A)	1.873(5)	Cu(3)-N(8A)#5	1.944(4)
Cu(4A)-Cu(6)#2	3.000(3)	Cu(3)-Cu(1B)#6	2.971(13)
Cu(4A)-Cu(2)#1	3.059(3)	Cu(5)-C(3B)#4	1.948(4)
N(1A)-C(1A)	1.157(5)	Cu(5)-N(3A)#4	1.948(4)
N(1A)-Cu(6)#3	1.942(3)	Cu(5)-Cu(1A)#1	2.913(4)
N(2A)-C(2A)	1.155(5)	Cu(5)-Cu(1B)#1	3.016(11)
N(2A)-Cu(2)	1.929(4)	Cu(6)-C(1B)#7	1.942(3)
C(3A)-N(3A)	1.158(5)	Cu(6)-N(1A)#7	1.942(3)
C(3A)-Cu(2)	1.923(4)	Cu(6)-Cu(4A)#2	3.000(3)
N(3A)-Cu(5)#4	1.948(4)	Cu(6)-Cu(4B)#2	3.004(10)
N(4A)-C(4A)	1.164(5)	C(9A)-N(9)	1.461(7)
N(4A)-Cu(2)	1.950(3)	C(9A)-H(9A1)	0.9800
C(4A)-Cu(3)	1.911(4)	C(9A)-H(9A2)	0.9800
C(5A)-N(5A)	1.162(5)	C(9A)-H(9A3)	0.9800
N(5A)-Cu(3)	1.931(3)	C(11A)-N(9)	1.527(8)
C(6A)-N(6A)	1.166(5)	C(11A)-H(11A)	0.9800
N(6A)-Cu(5)	1.926(3)	C(11A)-H(11B)	0.9800
N(7A)-C(7A)	1.173(5)	C(11A)-H(11C)	0.9800
N(7A)-Cu(6)	1.934(3)	C(9B)-N(9)	1.637(18)
C(7A)-Cu(5)	1.916(4)	C(9B)-H(9A)	0.9800
C(8A)-N(8A)	1.155(5)	C(9B)-H(9AB)	0.9800
C(8A)-Cu(6)	1.921(4)	C(9B)-H(9AC)	0.9800
N(8A)-Cu(3)#5	1.944(4)	C(11B)-N(9)	1.388(18)
Cu(1B)-N(2B)	1.784(9)	C(11B)-H(11D)	0.9800
Cu(1B)-N(1B)	1.930(10)	C(11B)-H(11E)	0.9800
Cu(1B)-Cu(3)#6	2.971(13)	C(11B)-H(11F)	0.9800
Cu(1B)-Cu(5)#1	3.016(11)	N(9)-C(12)	1.478(5)
Cu(4B)-N(6B)	1.770(10)	N(9)-C(10)	1.484(5)
Cu(4B)-N(5B)	1.933(12)	C(10)-H(10A)	0.9800
Cu(4B)-Cu(6)#2	3.004(10)	C(10)-H(10B)	0.9800
Cu(4B)-Cu(2)#1	3.015(10)	C(10)-H(10C)	0.9800
C(1B)-N(1B)	1.157(5)	C(12)-H(12A)	0.9800
C(1B)-Cu(6)#3	1.942(3)	C(12)-H(12B)	0.9800
C(2B)-N(2B)	1.155(5)	C(12)-H(12C)	0.9800
C(2B)-Cu(2)	1.929(4)	N(10)-C(13)	1.484(5)
N(3B)-C(3B)	1.158(5)	N(10)-C(15)	1.485(6)
N(3B)-Cu(2)	1.923(4)	N(10)-C(16)	1.486(5)
C(3B)-Cu(5)#4	1.948(4)	N(10)-C(14)	1.497(5)
C(4B)-N(4B)	1.164(5)	C(13)-H(13A)	0.9800
C(4B)-Cu(2)	1.950(3)	C(13)-H(13B)	0.9800
N(4B)-Cu(3)	1.911(4)	C(13)-H(13C)	0.9800
N(5B)-C(5B)	1.162(5)	C(14)-H(14A)	0.9800
C(5B)-Cu(3)	1.931(3)	C(14)-H(14B)	0.9800
N(6B)-C(6B)	1.166(5)	C(14)-H(14C)	0.9800
C(6B)-Cu(5)	1.926(3)	C(15)-H(15A)	0.9800
C(7B)-N(7B)	1.173(5)	C(15)-H(15B)	0.9800
C(7B)-Cu(6)	1.934(3)	C(15)-H(15C)	0.9800
N(7B)-Cu(5)	1.916(4)	C(16)-H(16A)	0.9800
N(8B)-C(8B)	1.155(5)	C(16)-H(16B)	0.9800
N(8B)-Cu(6)	1.921(4)	C(16)-H(16C)	0.9800

Table S6. Selected bond angles of **2** at 100 K.

C(1A)-Cu(1A)-C(2A)	174.7(4)	N(8B)-Cu(6)-C(7B)	119.57(14)

C(1A)-Cu(1A)-Cu(5)#1	102.7(2)	N(8B)-Cu(6)-C(1B)#7	123.83(15)
C(2A)-Cu(1A)-Cu(5)#1	81.40(15)	C(7B)-Cu(6)-C(1B)#7	116.24(14)
C(5A)-Cu(4A)-C(6A)	176.9(3)	C(8A)-Cu(6)-N(1A)#7	123.83(15)
C(5A)-Cu(4A)-Cu(6)#2	87.95(16)	N(7A)-Cu(6)-N(1A)#7	116.24(14)
C(6A)-Cu(4A)-Cu(6)#2	90.74(15)	C(8A)-Cu(6)-Cu(4A)#2	83.50(12)
C(5A)-Cu(4A)-Cu(2)#1	101.25(16)	N(7A)-Cu(6)-Cu(4A)#2	91.10(13)
C(6A)-Cu(4A)-Cu(2)#1	79.45(15)	N(1A)#7-Cu(6)-Cu(4A)#2	101.54(12)
Cu(6)#2-Cu(4A)-Cu(2)#1	164.99(17)	N(8B)-Cu(6)-Cu(4B)#2	86.0(3)
C(1A)-N(1A)-Cu(6)#3	171.5(3)	C(7B)-Cu(6)-Cu(4B)#2	87.1(4)
N(1A)-C(1A)-Cu(1A)	178.2(4)	C(1B)#7-Cu(6)-Cu(4B)#2	102.8(2)
C(2A)-N(2A)-Cu(2)	171.9(3)	N(9)-C(9A)-H(9A1)	109.5
N(2A)-C(2A)-Cu(1A)	176.6(4)	N(9)-C(9A)-H(9A2)	109.5
N(3A)-C(3A)-Cu(2)	172.3(3)	H(9A1)-C(9A)-H(9A2)	109.5
C(3A)-N(3A)-Cu(5)#4	171.9(3)	N(9)-C(9A)-H(9A3)	109.5
C(4A)-N(4A)-Cu(2)	170.8(3)	H(9A1)-C(9A)-H(9A3)	109.5
N(4A)-C(4A)-Cu(3)	172.3(3)	H(9A2)-C(9A)-H(9A3)	109.5
N(5A)-C(5A)-Cu(4A)	177.5(4)	N(9)-C(11A)-H(11A)	109.5
C(5A)-N(5A)-Cu(3)	173.5(3)	N(9)-C(11A)-H(11B)	109.5
N(6A)-C(6A)-Cu(4A)	177.8(4)	H(11A)-C(11A)-H(11B)	109.5
C(6A)-N(6A)-Cu(5)	171.7(3)	N(9)-C(11A)-H(11C)	109.5
C(7A)-N(7A)-Cu(6)	173.2(3)	H(11A)-C(11A)-H(11C)	109.5
N(7A)-C(7A)-Cu(5)	172.9(3)	H(11B)-C(11A)-H(11C)	109.5
N(8A)-C(8A)-Cu(6)	172.7(3)	N(9)-C(9B)-H(9A)	109.5
C(8A)-N(8A)-Cu(3)#5	172.1(3)	N(9)-C(9B)-H(9AB)	109.5
N(2B)-Cu(1B)-N(1B)	171.2(11)	H(9A)-C(9B)-H(9AB)	109.5
N(2B)-Cu(1B)-Cu(3)#6	95.0(5)	N(9)-C(9B)-H(9AC)	109.5
N(1B)-Cu(1B)-Cu(3)#6	89.2(4)	H(9A)-C(9B)-H(9AC)	109.5
N(2B)-Cu(1B)-Cu(5)#1	79.8(4)	H(9AB)-C(9B)-H(9AC)	109.5
N(1B)-Cu(1B)-Cu(5)#1	96.7(5)	N(9)-C(11B)-H(11D)	109.5
Cu(3)#6-Cu(1B)-Cu(5)#1	172.3(6)	N(9)-C(11B)-H(11E)	109.5
N(6B)-Cu(4B)-N(5B)	171.4(11)	H(11D)-C(11B)-H(11E)	109.5
N(6B)-Cu(4B)-Cu(6)#2	92.7(4)	N(9)-C(11B)-H(11F)	109.5
N(5B)-Cu(4B)-Cu(6)#2	85.9(4)	H(11D)-C(11B)-H(11F)	109.5
N(6B)-Cu(4B)-Cu(2)#1	82.2(4)	H(11E)-C(11B)-H(11F)	109.5
N(5B)-Cu(4B)-Cu(2)#1	100.0(4)	C(11B)-N(9)-C(12)	123.5(9)
Cu(6)#2-Cu(4B)-Cu(2)#1	172.7(6)	C(9A)-N(9)-C(12)	112.6(4)
N(1B)-C(1B)-Cu(6)#3	171.5(3)	C(11B)-N(9)-C(10)	117.8(8)
C(1B)-N(1B)-Cu(1B)	171.5(6)	C(9A)-N(9)-C(10)	115.3(4)
N(2B)-C(2B)-Cu(2)	171.9(3)	C(12)-N(9)-C(10)	110.4(3)
C(2B)-N(2B)-Cu(1B)	176.0(7)	C(9A)-N(9)-C(11A)	107.5(5)
C(3B)-N(3B)-Cu(2)	172.3(3)	C(12)-N(9)-C(11A)	105.7(4)
N(3B)-C(3B)-Cu(5)#4	171.9(3)	C(10)-N(9)-C(11A)	104.6(4)
N(4B)-C(4B)-Cu(2)	170.8(3)	C(11B)-N(9)-C(9B)	105.3(12)
C(4B)-N(4B)-Cu(3)	172.3(3)	C(12)-N(9)-C(9B)	96.7(7)
C(5B)-N(5B)-Cu(4B)	174.5(6)	C(10)-N(9)-C(9B)	96.1(8)
N(5B)-C(5B)-Cu(3)	173.5(3)	N(9)-C(10)-H(10A)	109.5
C(6B)-N(6B)-Cu(4B)	172.2(7)	N(9)-C(10)-H(10B)	109.5
N(6B)-C(6B)-Cu(5)	171.7(3)	H(10A)-C(10)-H(10B)	109.5
N(7B)-C(7B)-Cu(6)	173.2(3)	N(9)-C(10)-H(10C)	109.5
C(7B)-N(7B)-Cu(5)	172.9(3)	H(10A)-C(10)-H(10C)	109.5
C(8B)-N(8B)-Cu(6)	172.7(3)	H(10B)-C(10)-H(10C)	109.5
N(8B)-C(8B)-Cu(3)#5	172.1(3)	N(9)-C(12)-H(12A)	109.5
C(3A)-Cu(2)-N(2A)	125.09(15)	N(9)-C(12)-H(12B)	109.5
N(3B)-Cu(2)-C(2B)	125.09(15)	H(12A)-C(12)-H(12B)	109.5
N(3B)-Cu(2)-C(4B)	116.23(14)	N(9)-C(12)-H(12C)	109.5
C(2B)-Cu(2)-C(4B)	118.30(15)	H(12A)-C(12)-H(12C)	109.5
C(3A)-Cu(2)-N(4A)	116.23(14)	H(12B)-C(12)-H(12C)	109.5
N(2A)-Cu(2)-N(4A)	118.30(15)	C(13)-N(10)-C(15)	109.2(4)
N(3B)-Cu(2)-Cu(4B)#1	85.2(3)	C(13)-N(10)-C(16)	110.5(3)

C(2B)-Cu(2)-Cu(4B)#1	106.0(2)	C(15)-N(10)-C(16)	108.7(3)
C(4B)-Cu(2)-Cu(4B)#1	84.0(4)	C(13)-N(10)-C(14)	109.8(3)
C(3A)-Cu(2)-Cu(4A)#1	87.37(12)	C(15)-N(10)-C(14)	110.1(4)
N(2A)-Cu(2)-Cu(4A)#1	107.46(12)	C(16)-N(10)-C(14)	108.5(3)
N(4A)-Cu(2)-Cu(4A)#1	80.09(14)	N(10)-C(13)-H(13A)	109.5
N(4B)-Cu(3)-C(5B)	122.41(14)	N(10)-C(13)-H(13B)	109.5
C(4A)-Cu(3)-N(5A)	122.41(14)	H(13A)-C(13)-H(13B)	109.5
N(4B)-Cu(3)-C(8B)#5	119.29(14)	N(10)-C(13)-H(13C)	109.5
C(5B)-Cu(3)-C(8B)#5	117.95(14)	H(13A)-C(13)-H(13C)	109.5
C(4A)-Cu(3)-N(8A)#5	119.29(14)	H(13B)-C(13)-H(13C)	109.5
N(5A)-Cu(3)-N(8A)#5	117.95(14)	N(10)-C(14)-H(14A)	109.5
N(4B)-Cu(3)-Cu(1B)#6	87.9(3)	N(10)-C(14)-H(14B)	109.5
C(5B)-Cu(3)-Cu(1B)#6	102.0(2)	H(14A)-C(14)-H(14B)	109.5
C(8B)#5-Cu(3)-Cu(1B)#6	85.7(2)	N(10)-C(14)-H(14C)	109.5
C(7A)-Cu(5)-N(6A)	126.22(14)	H(14A)-C(14)-H(14C)	109.5
N(7B)-Cu(5)-C(6B)	126.22(14)	H(14B)-C(14)-H(14C)	109.5
N(7B)-Cu(5)-C(3B)#4	116.56(14)	N(10)-C(15)-H(15A)	109.5
C(6B)-Cu(5)-C(3B)#4	116.84(14)	N(10)-C(15)-H(15B)	109.5
C(7A)-Cu(5)-N(3A)#4	116.56(14)	H(15A)-C(15)-H(15B)	109.5
N(6A)-Cu(5)-N(3A)#4	116.84(14)	N(10)-C(15)-H(15C)	109.5
C(7A)-Cu(5)-Cu(1A)#1	78.50(14)	H(15A)-C(15)-H(15C)	109.5
N(6A)-Cu(5)-Cu(1A)#1	109.32(14)	H(15B)-C(15)-H(15C)	109.5
N(3A)#4-Cu(5)-Cu(1A)#1	87.88(12)	N(10)-C(16)-H(16A)	109.5
N(7B)-Cu(5)-Cu(1B)#1	82.6(3)	N(10)-C(16)-H(16B)	109.5
C(6B)-Cu(5)-Cu(1B)#1	106.4(2)	H(16A)-C(16)-H(16B)	109.5
C(3B)#4-Cu(5)-Cu(1B)#1	86.5(2)	N(10)-C(16)-H(16C)	109.5
C(8A)-Cu(6)-N(7A)	119.57(14)	H(16A)-C(16)-H(16C)	109.5
		H(16B)-C(16)-H(16C)	109.5

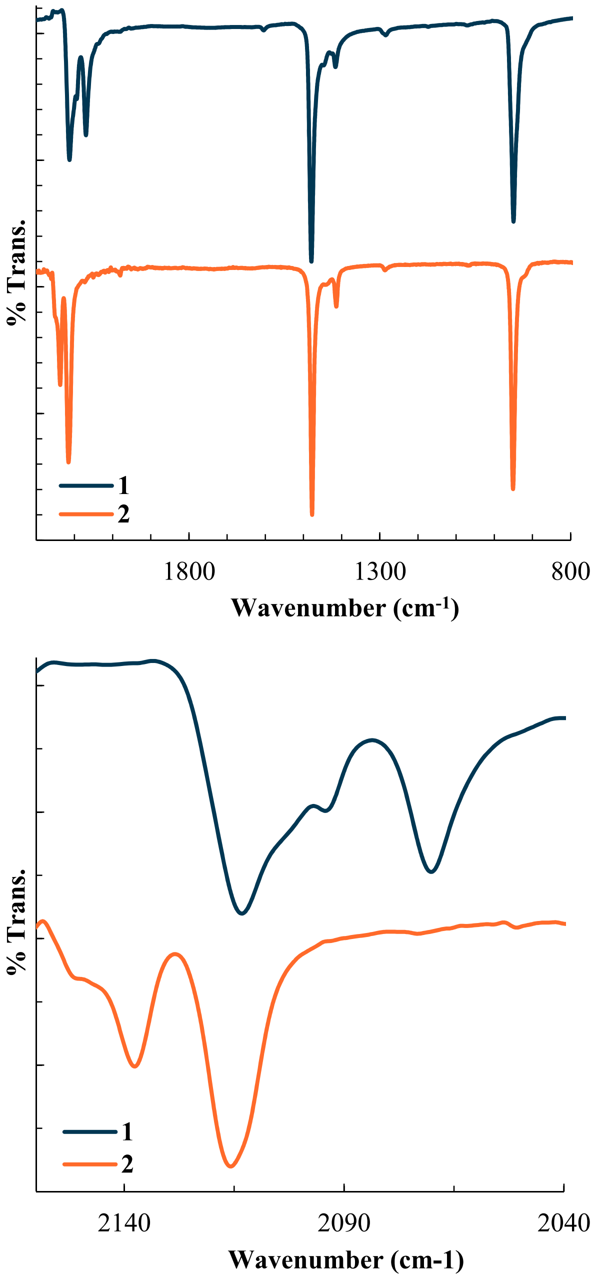

Figure S5. Vibrational infrared spectra of solid samples of **1** and **2** at 298 K.

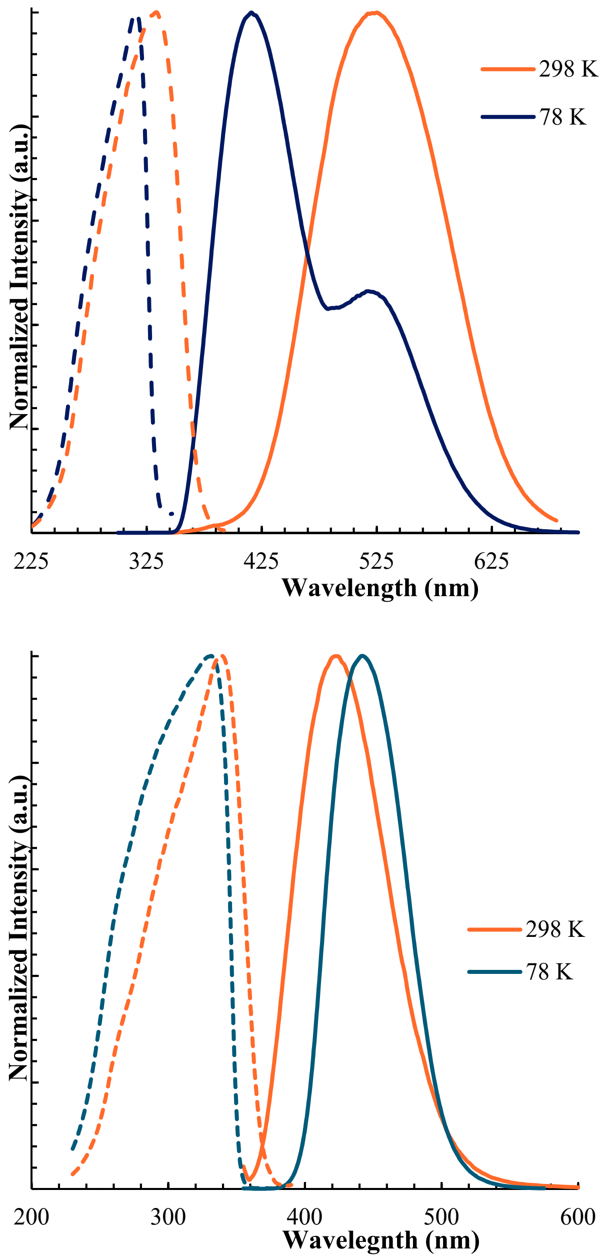

Figure S6. Luminescence spectrum of (left) **1** and (right) **2** at 78 K and 298 K.

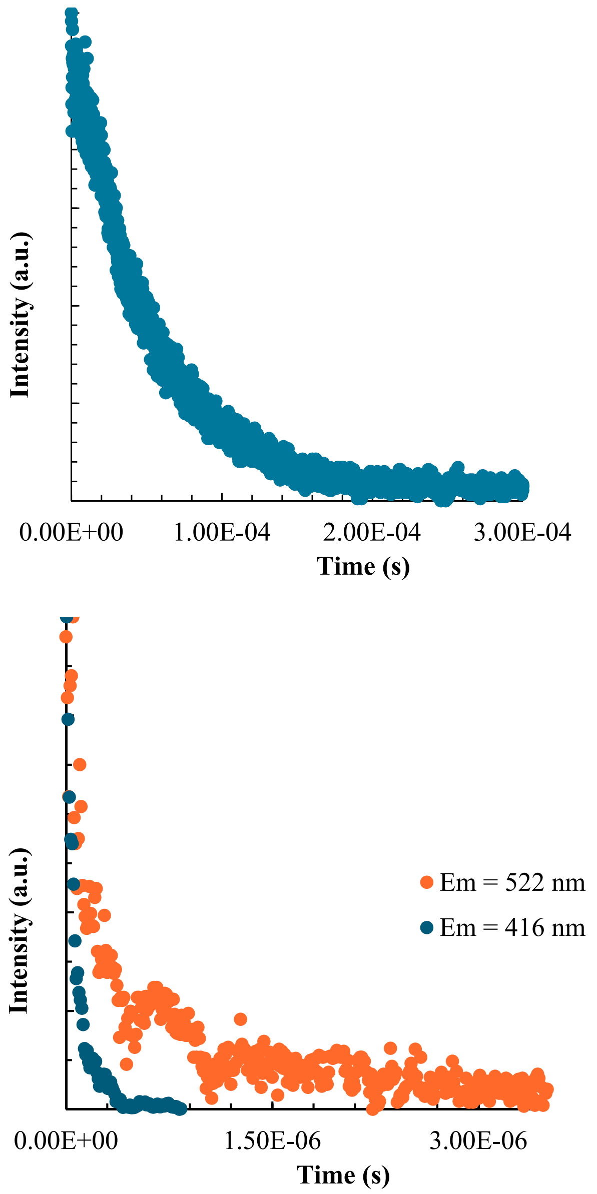

Figure S7. Lifetime measurements of **1** at (left) 298 K (λ_{em} = 522 nm) and (right) 78 K. An excitation wavelength of 350 nm was used for both lifetime measurements.

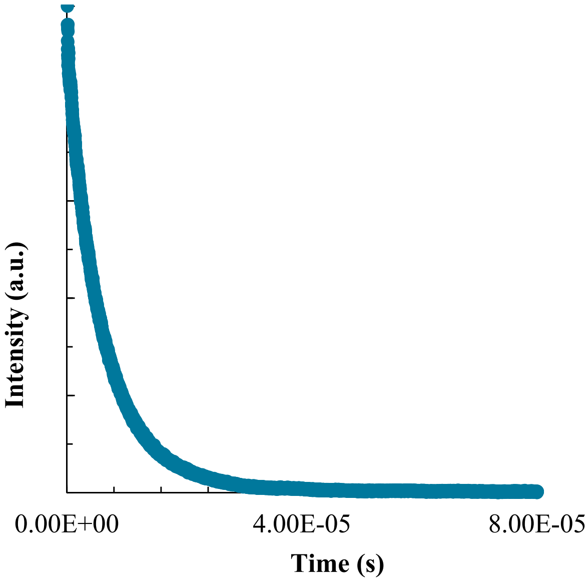

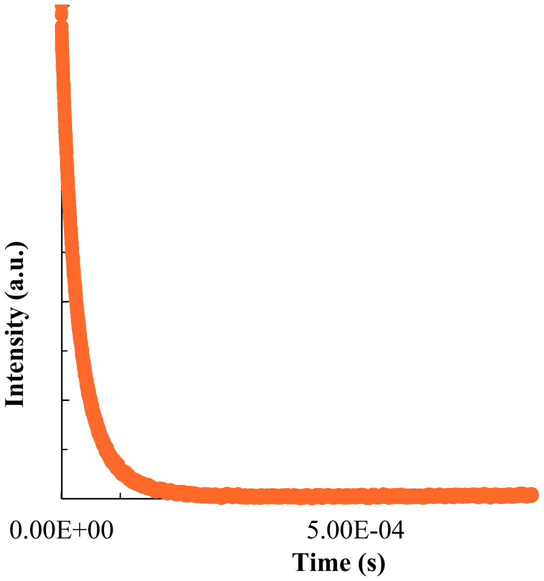

FigureS8. Lifetime measurements of **2** at (left) 298 K (λ_{em} = 420 nm) and (right) 78 K (λ_{em} = 440 nm). An excitation wavelength of 350 nm was used for both lifetime measurements.

Table S7. DFT ground state parameters of **1** compared to experimental values.

	Distances (Å)/Angles(°)		
	M06/CEP-31G(d)	B3LYP/SDD	Experimental
Cu···Cu (Rhomboid)	2.673	2.806	2.489
Cu_{C3v}···NMe_4^+	5.349	5.674	5.718
NC-Cu_{C3v}-CN	120.0	120.0	120.0
(μ^2-CN)-Cu-(μ^2-CN)	110.7	107.2	108.4

Table S8. DFT ground state parameters of **2** compared to experimental values.

	Distances (Å)/Angles(°)		
	M06/CEP-31G(d)	B3LYP/SDD	Experimental
Cu_{C3v}···Cu_{C3v}	5.127	5.116	4.980
Cu_{C3v}···$Cu_{C\infty v}$	5.223	5.166	4.926
$Cu_{C\infty v}$···NMe_4^+	4.935	5.510	5.141
Cu-Cu-Cu	166.1	172.0	175.4

NC-Cu$_{C3v}$-CN	107.3	107.6	118.5

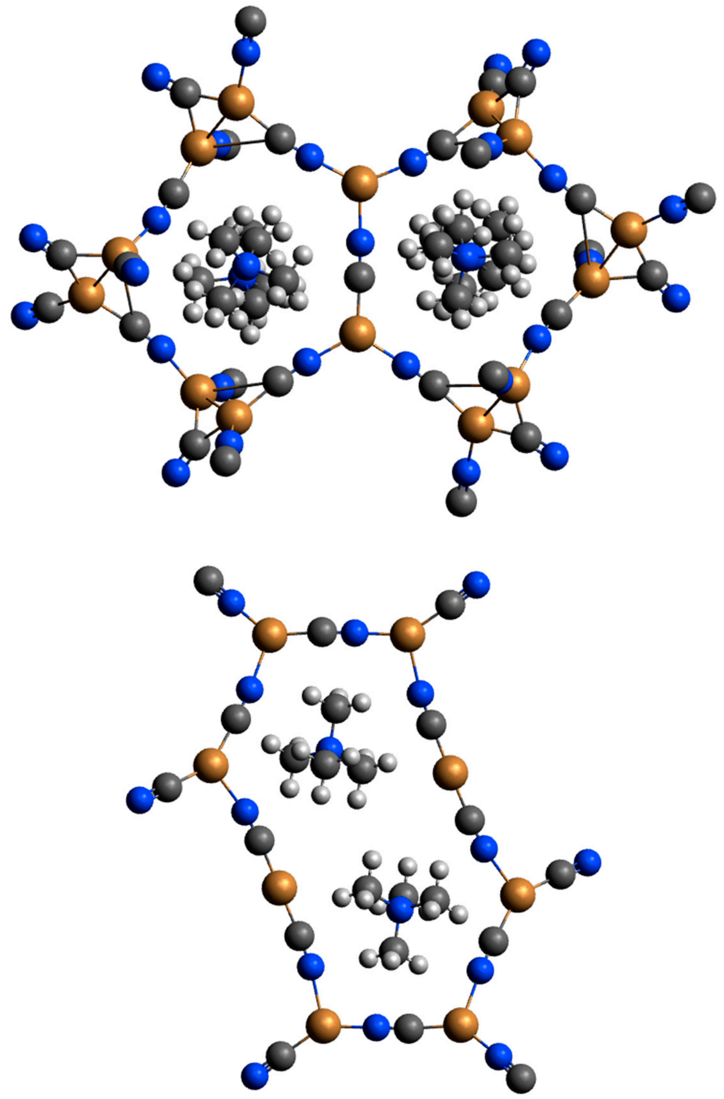

Figure S9. M06/CEP-31G(d) ground state structure of **1**(left) and **2** (right).

Table S9. M06/CEP-31G(d) TD-DFT calculation excited state transitions for **1** at 300 nm. Percent contributions noted.

Transition			Contribution	Percent Contribution
HOMO-5	→	LUMO+4	0.29692	27%
HOMO-5	→	LUMO	0.2794	24%
HOMO	→	LUMO	0.25509	20%
HOMO-8	→	LUMO+4	0.1319	5%
HOMO-5	→	LUMO+6	0.12917	5%
HOMO-5	→	LUMO+2	0.12013	4%
HOMO	→	LUMO+8	–0.10686	4%
HOMO	→	LUMO+5	0.10579	3%
HOMO	→	LUMO+1	0.10518	3%
HOMO-2	→	LUMO	–0.10325	3%

Table S10. M06/CEP-31G(d) TD-DFT calculation excited state transitions for **2** at 330 nm. Percent contributions noted.

Transition			Contribution	Percent Contribution
HOMO-8	→	LUMO	0.32687	24%

HOMO-9	→	LUMO	–0.2935	19%
HOMO-8	→	LUMO+1	–0.2126	10%
HOMO-4	→	LUMO+1	–0.1919	8%
HOMO-6	→	LUMO+1	–0.17759	7%
HOMO-6	→	LUMO+3	–0.14608	5%
HOMO-7	→	LUMO+1	0.14025	4%
HOMO-6	→	LUMO	0.13889	4%
HOMO-3	→	LUMO+1	–0.13586	4%
HOMO-9	→	LUMO+1	–0.13421	4%
HOMO-2	→	LUMO+1	–0.12752	4%
HOMO-7	→	LUMO	0.12265	3%
HOMO-6	→	LUMO+2	0.11918	3%

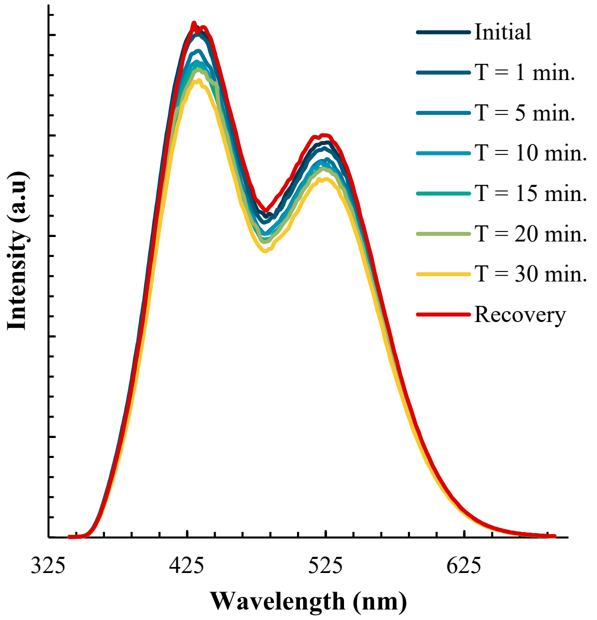

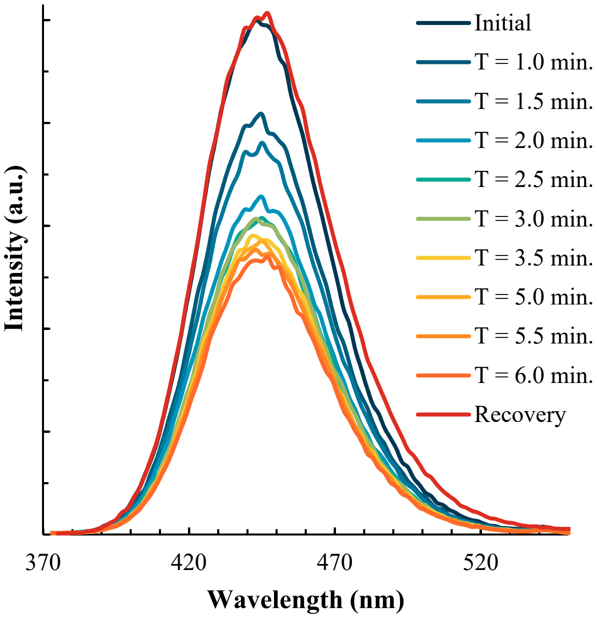

Figure S10. Uncorrected optical memory results of (left) **1** and (right) **2**.
